# Comprehensive Evaluation of the Expressed CD8+ T Cell Epitope Space Using High-Throughput Epitope Mapping

**DOI:** 10.3389/fimmu.2019.00655

**Published:** 2019-04-26

**Authors:** Paul V. Lehmann, Maneewan Suwansaard, Ting Zhang, Diana R. Roen, Greg A. Kirchenbaum, Alexey Y. Karulin, Alexander Lehmann, Pedro A. Reche

**Affiliations:** ^1^Cellular Technology Ltd., Shaker Heights, OH, United States; ^2^Laboratorio de Inmunomedicina & Inmunoinformatica, Departamento de Immunologia & O2, Facultad de Medicina, Universidad Complutense de Madrid, Madrid, Spain

**Keywords:** epitope, peptide, HCMV, EBV, MHC, HLA, T cell, ELISPOT

## Abstract

T cell immunity is traditionally assessed through functional recall assays, which detect the consequences of the T cells' antigen encounter, or via fluorescently labeled multimers that selectively bind peptide-specific T cell receptors. Using either approach, if the wrong antigen or peptide of a complex antigenic system, such as a virus, is used for immune monitoring, either false negative data will be obtained, or the magnitude of the antigen-specific T cell compartment will go largely underestimated. In this work, we show how selection of the “right” antigen or antigenic peptides is critical for successful T cell immune monitoring against human cytomegalovirus (HCMV). Specifically, we demonstrate that individual HCMV antigens, along with previously reported epitopes, frequently failed to detect CD8+ T cell immunity in test subjects. Through systematic assessment of T cell reactivity against individual nonamer peptides derived from the HCMVpp65 protein, our data clearly establish that (i) systematic testing against all potential epitopes encoded by the genome of the antigen of interest is required to reliably detect CD8+ T cell immunity, and (ii) genome-wide, large scale systematic testing of peptides has become feasible through high-throughput ELISPOT-based “brute force” epitope mapping.

## Introduction

Unlike B cells that endow humoral immunity through secretion of immunoglobulins, T cells exert their protective functions through direct interactions with antigen bearing cells. Conventional T cells recognize, via their T cell receptor (TCR), processed peptide fragments derived from polypeptide antigens, hereafter referred to as epitopes. These epitopes are displayed for T cell recognition on dedicated antigen-presenting molecules that are encoded by the major histocompatibility complex (MHC), which in humans is termed human leukocyte antigen complex (HLA). The TCR binds a binary complex of the epitope aligned in the peptide-binding groove of the MHC molecule (1, 2) displayed on the surface of antigen presenting cells (APC).

The TCR of the CD8+ subpopulation of T cells (CD8+ T cells) recognize epitopes presented in the context of MHC Class I molecules (MHC-I), while the TCR of CD4+ T cells recognize epitopes presented by Class II (MHC-II) molecules. During infection or vaccination, MHC molecules on APC will be loaded with peptide fragments derived from the individual proteins (antigens) that constitute the infectious agent (the antigen-system), and are transported to the APC's surface for T cell recognition (3). The specific peptide fragments from an antigen that are presented to T cells is primarily dictated by the MHC molecules encoded by an individual, and like the MHC molecules themselves, underlies considerable inter-individual variability. MHC I and II molecules are polygenic, and moreover each locus underlies extensive allelic polymorphism. Consequently, individuals in an outbred population will express a unique set of MHC molecules (4). As the polygenism and polymorphism of MHC primarily affects the peptide binding grove, each MHC locus and allele essentially encode for a distinct peptide binding motif, i.e., peptide binding specificity (5). While human subjects can share a common HLA-allele, there are few, if any, unrelated individuals on this planet who express the same constellation of HLA molecules. For this reason, it is extremely unlikely that two humans on this earth whose T cell system, when faced with the need to recognize a given infectious organism, would recognize an identical set of epitopes. The *potential epitope space*, that is, the array of peptides that meet MHC binding criteria in a given host is, therefore, unique to that individual. The highly individualized MHC-restricted recognition of epitopes by T cells is thought to have evolved to protect the species against antigenic mimicry: if a pathogen succeeds to evade immune recognition in one individual, or a subset of individuals sharing a certain MHC allele, it might endanger this subject or subpopulation, but not the species as a whole (6).

The T cell response elicited by an infection or immunization is dictated not only by the above inter-individual diversity of epitopes displayed for T cell recognition, but also by the inherent variability in the TCR repertoire possessed by different individuals. An individual's TCR repertoire is shaped by genetics, but also through selection during the development of self-tolerance, and by foreign antigens during environmental exposures or infections (7, 8). Due to such TCR repertoire variations, different individuals' T cells will not respond uniformly to the *potential epitope space*. For example, even if individuals share an HLA molecule, such as the HLA-A^*^02:01 allele, which should present the same set peptides, these individuals will not generate a uniform T cell response to these peptides (9). The array of epitopes actually recognized by T cells (constituting the *expressed T cell repertoire*) can therefore differ between individuals even if the potential epitope space (dictated by the MHC) is identical or overlapping between these individuals. The expressed T cell repertoire encompasses only a fraction of the expressed epitope space (10), but being dictated by the latter, will also be highly unique to each individual.

T cell immune monitoring aims at defining the expressed T cell repertoire, that is, the magnitude and quality of the antigen-specific T cells that have been generated in the course of the immune response. The magnitude of the expressed T cell repertoire is determined by the sum of all T cells that target all individual epitopes of the antigen system (e.g., virus) that have triggered a T cell response in the individual. Likewise, the quality of the elicited T cell response is defined by the effector functions of these T cells, such as the type of cytokine they secrete, or whether they are cytolytic. Given the multitude and variability of epitopes (peptides) of an antigenic system recognized in different individuals, a major challenge for T cell monitoring has been identification of the “relevant” epitopes (i.e., test peptides) for each test subject. In an attempt to address this, the mainstream approach has focused on one, or very few, epitopes restricted by an individual HLA allele and to study T cell recognition of peptides that have either been previously reported, or predicted, to be restricted by that allele due to its peptide binding motif (11). Owing to the high prevalence of the HLA-A^*^02:01 allele in the Caucasian population, immune monitoring has frequently focused on peptides restricted to this particular HLA-A allele. Approximately half of the Caucasian population expresses the HLA-A^*^02:01 allele (12), but it is less frequent in other races.

Although narrowing immune monitoring to single HLA-alleles is common, presently it is unclear how adequate this approach is. Any T cell immune monitoring effort that restricts itself to one or few epitopes presented by one HLA allele of a test subject inherently has the disadvantage of neglecting the remainder of HLA alleles expressed by the test subject. For example, selecting a single HLA Class I allele (one of up to six capable of presenting epitopes to CD8+ T cells in each test subject), and one test peptide capable of binding to the selected HLA molecule, can only be a valid approach to CD8+ T cell immune monitoring if CD8+ T cells indeed prevalently target that single epitope in the test individuals. Such immune dominance, however, might be the exception in humans, as we tend not only to respond to a multitude of epitopes of an antigen, but also with highly variable clonal sizes to the individual epitopes (9). Therefore, ideally, T cell immune monitoring should comprehensively include the entire potential epitope space, encompassing all peptides that could be restricted by all HLA alleles present in every test subject. This can be accomplished by using pools of such peptides, or by testing all peptides individually, in a “brute force” high-throughput approach (13–15). The former, while much simpler in scope, enables comprehensive assessment of the expressed antigen-specific T cell repertoire, but does not reveal the epitope specificity of the antigen-reactive T cells, whereas the latter also provides this valuable information.

The ability to perform high-throughput testing of hundreds, even tens of thousands of peptides, on individual test subjects has only recently become technologically feasible (16, 17). The hurdles that needed to be overcome included limitations in peripheral blood mononuclear cell (PBMC) numbers available from humans, ease of access to extensive custom peptide libraries, high-throughput T cell assay platforms, and automated data analysis including databasing. While such brute force epitope mapping seems to call for a major effort that can be undertaken only by industrial scale laboratories with essentially unlimited manpower and budgets, we have developed and report here high-throughput epitope mapping strategies that can be readily applied in even small academic laboratories operating on tight budgets. In this paper we outline how it can be implemented. Before doing so, using CD8+ T cell immunity to HCMV (and, Supplementary Material for EBV and influenza virus) as a model, we illustrate how incomplete our understanding of an individual's epitope space is to date, and thus how far we still are from comprehensive T cell immune monitoring. We also show the feasibility of systematic high-throughput (“brute force”) epitope scans as the necessary next step to fill this void.

## Materials and Methods

Peripheral blood mononuclear cells (PBMC) from healthy human donors (HD) were obtained from CTL's ePBMC library (CTL, Shaker Heights, OH, USA). The PBMC had been collected by HemaCare Blood Donor Center (Van Nuys, CA) under HemaCare's IRB and sold to CTL identifying donors by code only while concealing the subjects' identity. All PBMC were from healthy adults who had not taken medication within a month of the blood draw that might influence their T cell response. In addition, tests were done on each donor at HemaCare's CLIA certified laboratory to identify common infections: serological testing was done for Syphilis, Human cytomegalovirus (HCMV), Epstein Barr Virus (EBV), Hepatitis B, Hepatitis C, Human Immunodeficiency Virus, Human T-Lymphotropic Virus, and *Trypanosoma cruzi*. Subjects positive for any of these infections, except for HCMV and EBV were disqualified for the ePBMC library. The CMV and EBV serological status is specified for donors and cohorts in the manuscript. The donor's age, sex, ethnicity and HLA-type is shown in Supplementary Table 1. HLA-typing was done by the HLA Typing Laboratory of University of Oklahoma Health science Center (Oklahoma City, OK). For all experiments donor cohorts were selected according to the HCMV or EBV serostatus specified, and then tested in T cell assays with the T cell assay results shown. The cryopreserved cells were thawed following an optimized protocol (18) resulting in viability exceeding 90% for all samples. The PBMC were resuspended in CTL-Test™ Medium (from CTL). CTL-Test™ Medium is serum-free and has been developed for low background and high signal performance in ELISPOT assays. The number of PBMC plated in the different ELISPOT experiments varied between 2 and 4 × 10^5^ PBMC per well, with the number specified in Results for each experiment. The ELISPOT data are expressed as spot forming cells per million input PBMC (SFC/ 1 × 10^6^ PBMC).

### Antigens and Peptides

The peptide pools representing the HCMV antigens listed in Table 1, and EBV antigens specified in Supplementary Table 2, consisted of 15-mer peptides that covered the entire amino acid (aa) sequence of the respective proteins in steps (gaps of) 11 aa. Each of these peptide pools was purchased from JPT (Berlin, Germany), and were tested at a final concentration of 1 μg/mL in ELISPOT assays. The HCMV pp65_495−503_ peptide is an immunodominant peptide of HCMV that is presented by the HLA-A^*^02:01 allele (19). This HCMV pp65_495−503_ peptide, along with additional HCMV, EBV, and influenza virus peptides specified in **Figure 3**, Supplementary Figures 2, 3, respectively, were purchased from Panatecs (Heilbronn, Germany) at >95% purity according to the manufacturer's specification. Each of these purified, single peptides were tested at 1 μg/mL in ELISPOT assays. HCMV and EBV grade 2 antigens (UV-inactivated virions) were purchased from Microbix (Mississauga, Ontario, Canada) and tested at a final concentration of 30 μg/mL. CPI (from CTL) was used as a positive control in all experiments because unlike CEF peptides, CPI elicits T cell recall responses in all healthy donors (20). CEF is a pool of 32 immune dominant nonamer peptides derived from CMV, EBV and the influenza virus commonly used as a positive control for CD8+ T cell recall (20). CPI is a combination of protein antigens derived from CMV, influenza and parainfluenza viruses, and was used at a final concentration of 6 μg/mL in ELISPOT assays.

**Table 1:** HCMV-seropositive and -seronegative healthy human donors' reactivity to 20 HCMV antigens represented by peptide pools.

			**HCMV-seropositive donors**						**HCMV-seronegative donors**					
		**Donor ID#**	**64**	**99**	**137**	**182**	**193**	**194**	**7**	**65**	**141**	**151**	**158**	**218**
**HCMV antigens (No. of peptides per pool)**	Medium		28	5	20	48	0	3	8	5	8	3	18	0
	IE-1 (120)^a^		88	116	1,380	2,416	696	988	0	0	12	0	0	0
	IE-2 (143)^b^		16	72	52	92	244	24	36	0	0	0	4	0
	pp65 (138)^c^		532	1,412	1,272	216	1,092	340	0	4	0	0	12	4
	UL28 (92)^d^		8	228	16	684	32	520	0	0	0	0	0	4
	UL32 (260)^e^		16	2,320	504	80	380	100	8	0	0	0	0	4
	UL36 (117)^f^		17,772	192	24	936	348	124	4	0	0	4	0	0
	UL40 (53)^g^		0	4	24	76	20	8	0	0	0	0	24	4
	UL48-sub1 (229)^h^		8	8	16	36	4	20	0	0	0	0	0	0
	UL48-sub2 (229)^i^		100	16	36	112	24	4	4	0	4	0	8	0
	UL55 (224)^j^		2,340	104	2,308	2,328	1,628	48	4	0	4	0	4	0
	UL82(137)^k^		0	132	128	472	4	4	0	0	4	0	0	0
	UL94(84)^l^		148	0	52	2,492	724	20	0	12	0	0	12	4
	UL99(45)^m^		0	0	152	68	16	24	0	0	0	4	0	0
	UL103(60)^n^		96	96	52	80	4	24	4	0	0	0	4	0
	UL151(82)^o^		0	0	56	36	0	4	0	0	0	0	0	0
	UL153(67)^p^		8	36	136	68	24	20	0	0	0	0	4	0
	US3(44)^q^		12	0	48	1,152	12	12	0	0	8	0	0	0
	US24(123)^r^		0	1,468	68	2,172	672	92	8	0	0	0	0	0
	US29(113)^s^		4	0	40	64	0	12	0	0	0	0	12	0
	US32(43)^t^		4	0	128	104	12	24	0	0	4	0	0	0
	CPI		1,203	2,073	608	1,738	1,800	655	443	843	805	553	1,038	665

*Pools of 15-mer peptides were tested that span the polypeptide sequence of each specified HCMV protein in steps of 11 amino acids, with the number of peptides contained within each pool specified in parentheses. Media was used as negative control and CPI as the positive control. The results are expressed as SFU/ 1 × 10^6^ PBMC*.

a *lmmediate-Early protein 1 of HCMV*;

b *lmmediate-Early protein 2 of HCMV*;

c *65 kDa phosphoprotein (pp65) of HCMV*;

d *Uncharacterized protein UL28 of HCMV*;

e *Large structural phosphoprotein UL32 of HCMV*;

f *Uncharacterized protein UL36 of HCMV*;

g *Uncharacterized protein UL40 of HCMV*;

h *subpool of Deneddylase UL48 of HCMV*;

i *subpool of Deneddylase UL48 of HCMV*;

j *Envelope glycoprotein B (UL55) of HCMV*;

k *71 kDa phosphoprotein (UL82) of HCMV*;

l *Capsid-binding protein UL94 of HCMV*;

m *Tegument protein UL99 of HCMV*;

n *Protein UL103 of HCMV*;

o *0rf UL151 of HCMV*;

p *0rf UL153 of HCMV*;

q *Unique short US3 glycoprotein of HCMV*;

r *Tegument protein US24 of HCMV*;

s *Uncharacterized protein HHRF4 (US29) of HCMV*;

t *Uncharacterized protein HHRR7 (US32) of HCMV*.

The 553 nonamer peptides, spanning the entire HCMV pp65 sequence in steps of single aa were purchased from JPT as a FastTrack CD8 epitope library, and are hereafter referred to as pp65 9-mer peptides. Individual pp65 9-mer peptides were not subjected to further purification following their synthesis; however, individual peptides were verified and quantified by JPT using LC-MS. The average purity of the pp65 9-mer peptides was 56%, and the purity of individual 9-mer peptides that elicited CD8+ T cell recall responses are specified in Table 2 for each respective peptide. The pp65 9-mer peptides were delivered as lyophilized powder with ~25 μg of a single 9-mer peptide present in a designated well of a 96-well plate, and were distributed across six 96-well plates. Individual peptides were initially dissolved using 50 μL DMSO, followed by addition of 200 μL of CTL-Test Medium generating a “primary peptide stock solution” at 100 μg/mL with 20% v/v DMSO. From each of these wells, using a 96-well multichannel pipettor, a “secondary, 10X, peptide stock solution” was prepared, with peptides at 2 μg/mL. On the day of testing, 20 μL from each well was transferred “en block,” with a 96-well multi-channel pipettor into pre-coated ImmunoSpot® assay plates containing 80 μL CTL-Test Medium. Finally, 100 μL of PBMC (containing 3 × 10^5^ cells) in CTL-Test media was added “en block” to achieve a final peptide concentration of 0.2 μg/mL in the ELISPOT assay.

**Table 2:** CD8+ T cell epitopes of HCMV pp65 protein recognized in A^*^02:01-positive healthy donors as established by brute force epitope mapping.

**Donor ID**	**HLA Class 1**			**Control**			**Antigen-induced IFN-γ** **(SFU) per 1,000,000 cells**												
								**Peptides of pp65 9-mer library**												
	**A**	**B**	**C**	**Medium**	**CPI**	**pp65(15-mer pool)**	**pp65(495-503)^1^,^a^**	**pp65(495-503)^1^**	**pp65(97-105)^2^**	**pp65(116-125)^3^**	**pp65(141-149)^4^**	**pp65(144-152)^5^**	**pp65(175-183)^6^**	**pp65(203-211)^7^**	**pp65(221-229)^8^**	**pp65(324-332)^9^**	**pp65(325-333)^10^**	**pp65(417-425)^11^**	**pp65(418-426)^12^**
284	02:01 68:01	40:08 44:03	03:04 16:01	7	2,071	1,004	175	201	0	238	0	0	0	394	0	1,145	1,327	0	0
285	02:01 03:01	35:01 45:01	06:02 16:01	11	402	36	14	29	0	0	0	0	0	0	0	0	0	0	0
300	02:01 24:02	39:05 51:01	02:02 07:02	11	2,952	1,681	1,114	1,074	0	0	0	0	0	0	0	0	0	0	0
331	02:01 68:03	39:05 51:01	07:02 15:09	0	2,844	533	341	458	65	80	136	446	0	0	235	0	0	0	0
350	02:01 03:01	07:24 18:01	07:01 07:02	11	2,234	1,976	40	38	0	0	0	0	0	0	0	0	0	1,496	515

1 *NLVPMVATV (44.8%)*;

2 *PTGRSICPS (64.7%)*;

3 *LPLKMLNIP (64.7%)*;

4 *HLPVADAVI (80.3%)*;

5 *VADAVIHAS (54.2%)*;

6 *WKEPDVYYT (54.2%)*;

7 *ELVCSMENT (41.2%)*;

8 *DQYVKVYLE (43.7%)*;

9 *QQIFLEVQA (41.0%)*;

10 *QIFLEVQAI (44.1%)*;

11 *TPRVTGGGA (43.3%)*;

12 *PRVTGGGAM (40.1%)*.

a *More than 95.0% purity*.

SFU counts 1 × 10^6^ PBMC are shown only for peptides that elicited recall responses with peptides being identified by amino acid positions and sequence. Positive results were defined as antigen-triggered SFU counts that exceeded 5 SD of the medium control SFU counts (the latter calculated from a total of 18 medium control wells tested per donor on six plates). While all five donors shared the HLA-A^*^02:01 allele, the other HLA class I alleles expressed are also listed in the table as they could also serve as restriction elements for the peptide-specific CD8+ T cells.

### Human IFN-γ ELISPOT Assays

Single-color enzymatic ImmunoSpot® kits from CTL were used for the detection of IFN-γ producing cells. Test procedures followed the manufacturer's recommendations. In brief, antigens/peptides were plated at the specified concentrations into capture antibody-precoated ELISPOT assay plates in a volume of 100 μL per well, dissolved in CTL-Test Medium. The plates with the antigen were stored at 37°C in a CO_2_ incubator for less than an hour until the PBMC were ready for plating. The PBMC were added in the numbers specified (between 200,000 and 400,000 cells/well) in 100 μL CTL-Test Medium and cultured with the antigens/peptides for 24 h at 37°C and 9% CO_2_ in an incubator. After removal of the cells, addition of detection antibody, and enzymatic visualization of plate-bound cytokine, the plates were air-dried prior to scanning and counting of spot forming units (SFU). ELISPOT plates were analyzed using an ImmunoSpot® EpiScan™ Reader, by CTL. SFU were automatically calculated by the ImmunoSpot® Software for each stimulation condition using the Autogate™ function (15).

### Statistical Analysis

Purified peptides and protein antigens were tested in triplicate wells. For these, a paired one-tailed Student's *t*-test was performed to identify positive responses relative to the medium control wells. As ELISPOT counts follow Gaussian (normal) distribution among replicate wells, the use of parametric statistics was justified to identify positive and negative responses, respectively (21). A *p* < 0.05 was considered as the cut-off for positive responses induced by the purified peptides. The 553 individual peptides of the pp65 9-mer peptide library were tested in single wells. For these peptides, the threshold for a positive response was set at exceeding 5 SD of the mean SFU count detected in 18 replicate media control wells.

### HLA-Binding Predictions

We assessed peptide-HLA I presentation by predicting peptide-HLA I binding using HLA I allele specific profile motif matrices (22–24). We considered that a given peptide binds to a specific HLA I molecule when its binding score ranks within the top 3% percentile of the binding scores computed for 1,000 random 9-mer peptides (average amino acid composition of proteins in the SwissProt database).

## Results

### T Cells Target Multiple Antigens of HCMV

The genome of HCMV encodes multiple viral proteins, each of which could constitute a viable target antigen for T cell recognition. To this end, we tested 20 such HCMV antigens, specified in Table 1, for their ability to recall T cell responses in healthy human donors. Peripheral blood mononuclear cells (PBMC) obtained from six HCMV-seropositive and six HCMV-seronegative human subjects were challenged for 24 h with the specified HCMV antigens to selectively stimulate the respective antigen-specific T cell populations to secrete IFN-γ. IFN-γ production was measured in a standard ELISPOT assay format in which the cytokine is captured on the membrane around the cells that secrete it, permitting the visualization and quantification of individual IFN-γ-secreting T cells as “spot forming units” (SFU). Thus, this assay measures, at a single-cell level, the number of T cells that engaged in IFN-γ production following antigen stimulation (25). The individual HCMV antigens used for stimulation were 15 amino acid (aa) long peptides that collectively spanned the respective polypeptide sequences in steps of (skipping) 11 aa, hereafter referred to as peptide pools. Each peptide was present at ~1 μg/mL within the respective peptide pools, and the number of peptides contained in each pool is specified in Table 1.

Stimulation of all six HCMV-seronegative donors with each of the twenty HCMV peptide pools failed to elicit an increased number of IFN-γ-producing T cells relative to PBMC cultured in media alone (Table 1). However, each of these HCMV-seronegative donor PBMC robustly responded to a combination of cytomegalovirus (C), parainfluenza (P), and influenza (I) antigens, collectively referred to as CPI (20), which confirmed T cell functionality in the respective samples (Table 1). The inability to detect a recall response to the HCMV peptide pools in HCMV-seronegative donors, in the face of their CPI reactivity, establishes the exquisite specificity of the HCMV peptide pool-triggered recall responses.

Stimulation of all six HCMV-seropositive donors' PBMC, in contrast, revealed recall responses to several of these HCMV antigens (Table 1). T cells specific for IE-1, pp65, and UL55 were detected in all six HCMV-seropositive donors, but the magnitude of recall responses was variable between donors, and varied also within a donor, ranging from relatively low SFU counts (in the tens) to high counts (in the hundreds). As the peptide pools tested on all donors were the same, and these were tested in a single experiment, the variability of responses observed must lie in the T cell compartment itself. There was no apparent response hierarchy seen for IE-1, pp65, and UL55. The IE-2, UL28, UL32, UL36, UL82, UL94, UL103, UL153, and US3 peptide pools also elicited recall responses in at least half of these donors, and again there was no clear response hierarchy seen against these antigens. For example, Donor 64 exhibited a high frequency recall response to both the UL36 and UL55 peptide pools, with negligible responses against several other peptide pools. In contrast, the response against pp65, UL32, and US3 prevailed in Donor 99. In aggregate, IE-1, pp65, UL28, UL32, UL36, UL55, UL94, US3, and US24 peptide pools were preferentially recognized by effector T cells from these HCMV-seropositive donors. Importantly, these antigens were not consistently immunodominant, whereas IE-2, UL40, UL48-1/2, UL99, UL103, UL151, UL153, US29, and US32 were either not recognized, or recalled a low frequency of T cells in the test subjects.

Collectively, the data presented in Table 1 demonstrate that for monitoring T cell immunity to HCMV one should not focus on a single viral antigen. Even if an antigen is immune dominant in a subset of donors, it might be subdominant or even negative in other donors who respond strongly to other antigens of the virus. Selecting one antigen over another may not only grossly underestimate the magnitude of the expressed T cell repertoire, but can also misrepresent it entirely. For example, pp65 was co-dominant with UL32 and US3 in Donor 99, whereas pp65 was co-dominant with IE-1 and UL55 in Donor 137. However, pp65 can also be a subdominant antigen. Such was the case for Donor 182, in whom the number of T cells responsive to IE-1, UL55, UL94, and US3 were each 10 times higher than those targeting pp65. Therefore, if pp65 reactivity would be used in this particular donor to assess the magnitude of the HCMV-specific T cell repertoire, one would detect only 1.6% of the HCMV antigen-specific T cells responsive to the epitope space covered by the 20 HCMV antigens used for testing. For HCMV, therefore, these data highlight the need to comprehensively test all antigens of the virus to reliably assess the magnitude of the anti-viral T cell response. This notion might apply to other viruses as well, since similar observations were made through testing T cell recognition of peptide libraries representing several EBV antigens (Supplementary Table 2).

### CD8+ T Cell Recognition of HCMV Epitope pp65(495-503) in HLA-A^*^02:01-Positive Test Subjects

**Figure 1:**
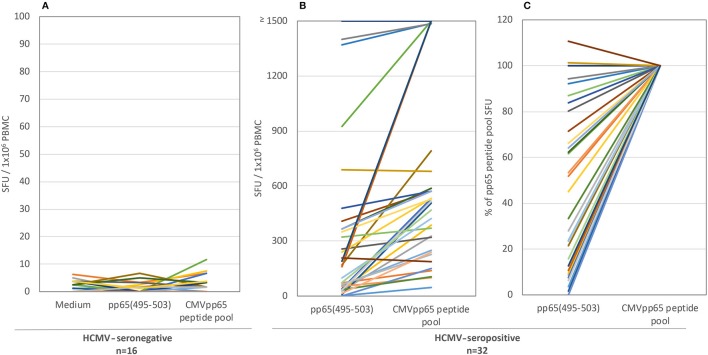
The T cell recall response to single peptide pp65_495−503_ vs. the pp65 peptide pool in HLA-A^*^02:01-positive test subjects. Both antigens were tested in an IFN-γ ELISPOT assay on 16 HCMV-seronegative subjects **(A)** and 32 HCMV-seropositive donors **(B)**. The results are expressed as SFU/ 1 × 10^6^ PBMC. Colored lines link each test subject's response to the two antigens. Panel **(C)** shows the normalized data for the seropositive cohort, with the SFU counts elicited by the pp65 peptide pool set as 100% for each donor, to which the magnitude of the pp65_495−503_ triggered SFU counts in the same donor was compared.

T cell immune monitoring is frequently performed using single peptides. Specifically, the NLVPMVATV peptide, corresponding to amino acids 495-503 of HCMV's pp65 protein (pp65_495−503_) has been described as an immune dominant epitope of HCMV in HLA-A^*^02:01-positive subjects (19). Having established that restricting immune monitoring to only the pp65 antigen might be insufficient, we next sought to establish how reliably restricting immune monitoring to a single pp65 peptide would reflect on the entire pp65 epitope space. As one approach to address this question, we tested 32 HLA-A^*^02:01-positive, HCMV-seropositive healthy human donors for reactivity against the single pp65_495−503_ peptide and the pp65 peptide library in parallel using an IFN-γ ELISPOT assay (Figure 1; the raw data are shown in Supplementary Table 3 permitting the identification of individual donors). As controls, PBMC from sixteen HLA-A^*^02:01-positive, HCMV-seronegative donors were tested in the same manner. Each of these control subjects' PBMC failed to respond to either antigen (Figure 1A), while exhibiting a strong recall response to CPI (data not shown). The absence of a recall response to the pp65_495−503_ peptide, and the pp65 peptide pool, in HCMV-seronegative donors serves to further support the specificity of the recall response these antigens elicited in HCMV-seropositive individuals.

**Figure 2:**
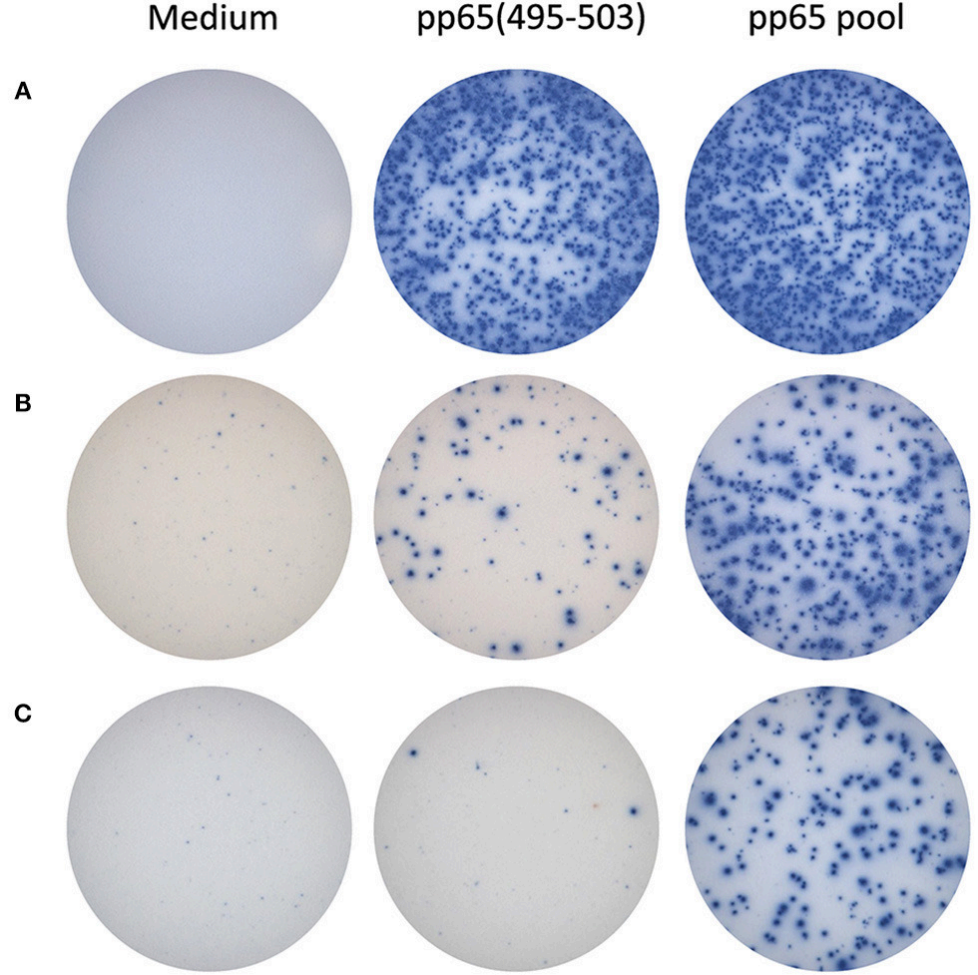
Representative raw data for pp65_495−503_ single peptide-reactive CD8+ cells vs. pp65 peptide pool-specific T cells in HLA A^*^02:01-positive donors. In row **(A)**, IFN-γ ELISPOT well images are shown for a donor with matching SFU counts: pp65_495−503_ appears to be immune dominant in this donor. In the donor shown in row **(B)**, pp65_495−503_ single-peptide reactive CD8+ T cells constitute a fraction of the pp65 peptide pool-reactive T cells. The donor shown in row **(C)** did not display pp65_495−503_ single-peptide reactive CD8+ T cells, although a response to the pp65 peptide pool was observed: in this donor apparently, other epitopes are targeted as well by CD8+ T cells.

For the HLA-A^*^02:01-positive, HCMV-seropositive donor cohort (*n* = 32), the number of T cells responsive to either the pp65_495−503_ peptide or pp65 peptide pool was determined (Figure 1B). Since our objective was to address whether the pp65_495−503_ peptide was indeed immunodominant within the epitope space of the pp65 antigen, we normalized the number of T cells activated by the pp65 peptide pool to 100% and expressed the number of pp65_495−503_ peptide reactive T cells as a percentage of this total (Figure 1C). Using 50% as an arbitrary cut-off for immunodominance, 15 of these 32 (47%) donors exhibited a dominant response to the pp65_495−503_ peptide. Therefore, in approximately half of HCMV-seropositive HLA-A^*^02:01 subjects, reactivity to the pp65_495−503_ peptide accurately reflected the expressed T cell repertoire against the pp65 antigen. However, in 17 of the remaining HCMV-seropositive HLA-A^*^02:01 subjects (53%), reactivity against the pp65_495−503_ peptide constituted <50% of the pp65 peptide pool-specific T cell repertoire. In these donors, it was therefore likely that additional epitopes covered by the pp65 peptide pool were also targeted, and that these might even outnumber the pp65_495−503_ -specific T cells. Notably, in six of these HLA-A^*^02:01-positive, HCMV-seropositive subjects (19%), the pp65_495−503_ peptide failed to recall detectable numbers of T cells whilst responses against the pp65 peptide pool were detected. In such donors, immune monitoring with the single pp65_495−503_ peptide, but not with the pp65 peptide pool, would yield false negative results since the expressed T cell repertoire targeted other regions of the pp65 protein. To illustrate this point further using raw data, in Figure 2 we present representative well images of donors for which the pp65_495−503_ peptide was immune dominant, encompassing nearly the entire epitope space covered by the pp65 peptide pool (Donor 261 in Row A of Figure 2). In contrast, a donor in whom pp65_495−503_ specific CD8+ T cells cannot be detected in spite of this donor's reactivity to the pp65 peptide pool (Donor 183 in Row C of Figure 2), and a donor for which the pp65_495−503_ epitope covers only a fraction of pp65's epitope space (Donor 213 in Row B of Figure 2) are also presented.

Overall, Figures 1, 2 establish that even in those rare cases in which immune dominance of a single peptide can occur, such as pp65_495−503_ peptide recognition in HLA-A^*^02:01-positive subjects, the expressed antigen-specific T cell repertoire can be frequently underestimated by this single peptide, even to the point of obtaining false negative data. Furthermore, immune monitoring of T cell responsiveness to a single antigen, or peptide thereof, would fail to capture the extent to which other antigens of the same antigen system were targeted (see Table 1).

For the above comparisons of T cell recognition of the pp65_495−503_ peptide *vs*. the entire pp65 epitope space, it must be noted that the numbers of pp65_495−503_ peptide-specific CD8+ cells can be expected to be accurate, whereas the numbers obtained using the pp65 peptide pool are likely to underrepresent the entire expressed pp65 antigen-specific T cell repertoire. The pp65_495−503_ peptide is HLA-A^*^02:01-restricted and too short for presentation by HLA class II molecules, hence it should exclusively elicit CD8+ T cells. In contrast, the pp65 peptide pool, consisting of 15-mer peptides, recalls both CD4+ and CD8+ T cells (Supplementary Figure 1). Fifteen-mer peptides can trigger, but are not ideal for, CD8+ T cell activation as the peptide binding groove of HLA class I molecules is closed on both ends and therefore accommodates only peptides of 9–11 aa, and moreover is intolerant to frame shifts of the peptide binding motif (11). The 15-mer peptides therefore need to undergo further processing to generate peptides 9-10 aa in length that are suitable for binding HLA I molecules, and such processing can also destroy these peptides. Moreover, as the peptides in the pp65 pool walk the pp65 sequence in steps of 11 aa, considerable gaps in epitope coverage can be expected. The possibility that the 15-mer peptide pool insufficiently covers the entire CD8+ T cell epitope space of pp65 suggests that this pool detects only a fraction of the antigen-specific CD8+ T cell repertoire. Therefore, the difference between the single peptide data, and the full antigen-specific CD8+ T cell repertoire is potentially even larger than suggested by the data shown in Figure 1. To this end, for comprehensive assessment of complete CD8+ cell epitope coverage of an antigen, it would be ideal to test peptides that are 9 to 10 aa long and that cover the protein sequence in steps of single amino acids, an approach we have undertaken below.

### CD8+ T Cell Recognition of HCMV Epitopes in HLA-B35-, B44-, B7-, and B18-Positive Subjects

**Figure 3:**
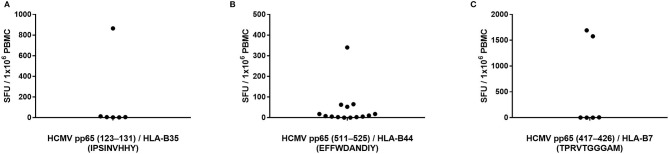
Previously reported HCMV epitopes frequently do not recall a CD8+ T cell response in HCMV-seropositive individuals expressing the matching HLA class I allele. The specified peptides were tested in an IFN-γ ELISPOT assay on PBMC of HCMV-seropositive donors expressing HLA-B35 **(A)**, HLA-B44 **(B)** or HLA-B7 **(C)**.

Peptide pp65_123−131_ (IPSINVHHY) is HLA-B35-restricted and has been described as an epitope frequently targeted by CD8+ T cells from HCMV-infected donors bearing this HLA allele (20). We identified seven HLA-B35-positive, HCMV-seropositive, and pp65 peptide pool-reactive donors and tested these donors' PBMC for T cell reactivity to this peptide. As shown in Figure 3A (the raw data are shown in Supplementary Table 4 permitting the identification of individual donors), only one of these seven donors' PBMC harbored pp65_123−131_-specific T cells in high numbers. In the other six donors tested, the frequency of pp65_123−131_-specific T cells was below the limit of detection for the assay, that is, <1 in 400,000 PBMC.

To further test whether single peptides are insufficient for accurate T cell immune monitoring, we assessed T cell reactivity against three additional pp65-derived peptides that are restricted by HLA-B alleles. The HLA-B44-restricted pp65_511−525_ (EFFWDANDIY) peptide has previously been reported as a prevalent epitope targeted by HCMV-infected individuals expressing this HLA allele (26). We identified 15 PBMC donors who were HLA-B44-positive, HCMV-seropositive, and also reacted strongly to the pp65 peptide pool. Only one of these donors exhibited a strong recall response to the pp65_511−525_ peptide (Figure 3B; the raw data are shown in Supplementary Table 4 permitting the identification of individual donors). Three additional HLA-B44-positive donors responded weakly to the pp65_511−525_ peptide, while we failed to measure a detectable response in the remaining 11 donors (73%). The pp65_417−426_ (TPRVTGGGAM) peptide is an HLA-B7-restricted epitope that has also been implicated as a prevalently recognized HCMVpp65 epitope (27). We had accessed to PBMC from six individuals who were HLA-B7-positive, HCMV-seropositive, and pp65 peptide pool-reactive. As shown in Figure 3C (the raw data are shown in Supplementary Table 4 permitting the identification of individual donors), only two of these donors (33%) possessed pp65_417−426_-specific T cells in high numbers, while in the remaining four donors, pp65_417−426_-specific T cells were undetectable in spite of their HCMV-positive status. Lastly, the pp65_378−389_ (SDEEEAIVAYTL) peptide is HLA-B18-restricted and has been described as a frequently targeted epitope in HCMV-infected donors who express this HLA allele (19). We had access to three HLA-B18-positive donors who were also HCMV-seropositive and responded to the pp65 peptide pool: none of these donors displayed a recall response to the pp65_378−389_ peptide (data not shown).

Supporting the notion put forward for the pp65_495−503_ epitope in Figure 1, the data presented in Figure 3 offer further support using three additional peptides previously described as “immune dominant” epitopes: such peptides were not necessarily targeted by T cells, and thus would frequently provide false negative results, or underestimate the HCMV-specific T cell repertoire. Instead, T cells frequently recognized alternative epitopes from the pp65 antigen, as suggested by the single-peptide negative donors' responsiveness to the pp65 peptide pool. Furthermore, pp65 is only one of many HCMV antigens recognized by the expressed T cell repertoire, and is not immunodominant in all HCMV-seropositive subjects (Table 1). Immune monitoring, therefore, that relies on single HCMV peptides is likely to provide false negative results in a considerable fraction of test subjects, and when peptide-reactive T cells are detected, their numbers might not provide an accurate reflection of the overall magnitude of the antigen-specific T cell repertoire in the test subject. We have made similar observations using the EBV and influenza test systems (Supplementary Figures 2, 3), suggesting that this notion might hold for T cell immunity to other viruses as well.

### Brute Force Epitope Mapping of HCMV pp65 Antigen

The frequent discordance between the frequency of CD8+ T cells specific for the pp65_495−503_ peptide vs. the pp65 peptide pool in HLA-A^*^02:01-positive, HCMV-seropositive subjects (Figure 1), along with the notion that the recall response triggered by the pp65 15-mer peptide pool encompasses both a CD8+ and CD4+ T cell component (Supplementary Figure 1) suggested that pp65_495−503_ might not be the sole immune dominant epitope in these subjects. In particular, we hypothesized that this was the case for donors in which the pp65_495−503_ peptide-induced recall response was weak relative to that triggered by the pp65 peptide pool (e.g., Donors 213 and 183 in Figure 2). To directly address this hypothesis, we set out to systematically identify all MHC class I-restricted T cell epitopes in the pp65 protein that were recognized using a brute force epitope mapping approach. Therefore, a series of 9-mer peptides was synthesized that span the entire sequence of the pp65 protein progressing in steps of a single amino acid (Supplementary Figure 4). These individual peptides were plated at 0.2 μg/mL, one peptide sequence per well, with the 553 unique peptides spanning across six 96-well plates for each test subject. Each plate contained 3 medium only control wells, and one well allocated for a CPI positive control. In the same experiment, each donor's PBMC (at 3 × 10^5^ cells/well) were also tested for their recall response to the pp65 15-mer peptide pool and to pp65_495−503_ peptide (from a different synthesis, at >95% purity). Of note, the individual peptides of the 9-mer library were not subjected to further purification after their synthesis, and averaged 56% purity. The results of the IFN-γ ELISPOT assays using the individual peptides of the pp65 9-mer series, along with controls, for stimulation of four HLA-A^*^02:01-positive, HCMV-seropositive healthy donors (Donors 284, 300, 331 and 350) and a single HLA-A^*^02:01-positive HCMV-seronegative donor (Donor 285) are summarized in Table 2.

As seen in Table 2, Donor 300 responded vigorously to the 15-mer pp65 peptide pool (1,681 SFU/ 1 × 10^6^ PBMC), and with a similar frequency to purified pp65_495−503_ peptide (1,114 SFU/1 × 10^6^ PBMC PBMC). The magnitude of the recall response to the corresponding (unpurified) pp65_495−503_ peptide from the peptide series was 1,074 SFU/1 × 10^6^ PBMC, which was essentially identical to the purified pp65_495−503_ peptide. Since the recall response induced by the purified and unpurified pp65_495−503_ peptides provided highly similar SFU counts for all donors tested, this result suggested that the 9-mer pp65 peptide series was well-suited for assessing CD8+ cell activation despite the absence of further purification following their synthesis. Unlike other donors, Donor 300 failed to exhibit a response to any of the other peptide in the 9-mer pp65 peptide series. Therefore, the expressed T cell repertoire of Donor 300 was indeed exclusively targeting the HLA-A^*^02:01-restricted pp65_495−503_ peptide.

For Donor 284, the pp65_495−503_ peptide-elicited recall response was 175 SFU/1 × 10^6^ PBMC for the purified peptide, and 60 SFU/300,000 PBMC for the corresponding 9-mer in the pp65 peptide series. Therefore, the response against the pp65_495−503_ peptide constituted only ~20% of the observed pp65 peptide pool response (1,004 SFU/1 × 10^6^ PBMC). In agreement with this observation, Donor 284 demonstrated recall responses to several additional 9-mers in the pp65 peptide series. Specifically, the pp65_116−125_ (238 SFU/1 × 10^6^ PBMC), pp65_203−211_ (394 SFU/1 × 10^6^ PBMC), pp65_324−332_ (1,145 SFU/1 × 10^6^ PBMC) and pp65_325−333_ (1,327 SFU/1 × 10^6^ PBMC) peptides all stimulated IFN-γ secretion by this donor's PBMC. Supplementary Figure 5 depicts raw data (well images) of the ELISPOT plate in which adjacent pp65_324−332_ and pp65_325−333_ peptides triggered a recall response in this donor. Being representative of results obtained from all plates, these well images are shown to illustrate the clarity of peptide-triggered signal over the negligible background noise in these epitope scanning assays. Since the pp65_324−332_ and pp65_325−333_ peptides are adjacent, the observed responses likely identify a single naturally processed CD8+ T cell epitope for this donor. Therefore, the peptide pp65_495−503_ was only one of 4 co-dominant epitopes recognized by CD8 T cells from Donor 284. Likewise, Donor 331 followed a similar pattern and recognized six distinct pp65 epitopes, of which five were identified by brute force epitope scanning and were recognized in a co-dominant fashion.

In Donor 350, the pp65_495−503_ peptide elicited recall response (40 SFU/1 × 10^6^ PBMC for the purified peptide, and 38 SFU/1 × 10^6^ PBMC PBMC for the corresponding nonamer from the 9-mer series) was only 2% of that triggered by the pp65 peptide pool (1,976 SFU/1 × 10^6^ PBMC). For this particular donor, two adjacent peptides, pp65_417−425_ and pp65_418−426_, that likely jointly reveal a single epitope, triggered a vigorous recall response with 1,496 and 513 SFU/1 × 10^6^ PBMC, respectively. No other peptide, except for the weak recall response to pp65_495−503_ itself, triggered SFU counts above the medium background (Table 2). Therefore, for Donor 350 the immune dominant epitope was pp65_417−425_/pp65_418−426_, with the pp65_495−503_ peptide contributing only minimally to CD8+ T cell recognition of the pp65 antigen.

Unlike the above HCMV-seropositive donors, the HCMV-seronegative Donor 285 failed to mount a significant response to the pp65 peptide pool or pp65_495−503_ peptide. This donor also did not yield a positive response to any of the peptides in the pp65 9-mer peptide series, supporting the specificity of responses induced by such peptides in Donors 284, 300, 331 and 350.

Overall, these brute force CD8+ epitope scanning experiments confirm the notion that immune monitoring that relies exclusively on a single “immune dominant” peptide can largely underestimate (Donors 284 and 331), or fail to detect (Donor 350) the antigen-specific CD8+ T cell repertoire. Only in one of four instances was the single peptide approach sufficient to accurately monitor T cell reactivity against a complex antigen (Donor 300). Therefore, to comprehensively assess the magnitude of antigen-specific CD8+ T cell immunity in a test subject population, it would be advisable to either test peptide pools or cover the entire epitope space using single peptides.

### Predicted vs. Actually Recognized pp65 Epitopes

**Figure 4:**
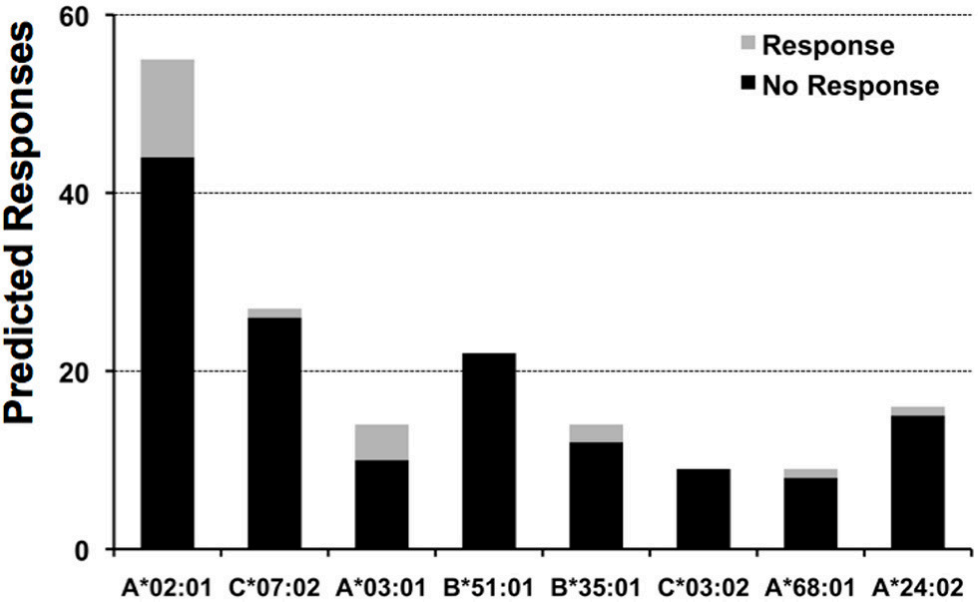
Predicted responses for selected HLA I molecules. The figure displays the number of predicted responses for 8 distinct HLA I molecules expressed by the test subjects included in this study. For each predicted response, we distinguished those that actually gave a recall response from those that did not give any response, the gray and black sections in column bars, respectively. The number of subjects that expressed the HLA I molecules specified are: A^*^02:01, 5 subjects; C^*^07:02, 3 subjects; A^*^03:01 & B^*^51:01, 2 subjects; B^*^35:01, C^*^03:04; A^*^68:01 and A^*^24:02, 1 subject.

The epitope space evaluated for pp65 consisted of 553 peptides, 9 aa in length, spanning the entire length of the protein with overlaps of 8 aa residues. We selected 9-mer peptides since the HLA I molecules have a preference for binding peptides of that size. We tested the response of 5 subjects to each of these peptides, identifying the high frequency responses shown in Table 2, plus 194 weaker responses, that is, SFU counts that exceeded the mean of the 18 medium control wells by 5 SD. We assessed how many of these responses could be anticipated by determining if peptides could be predicted to bind to any of the HLA I alleles expressed by the subjects (details in Material and Methods). Of note, we would have only predicted 20 of these responses, which constitute only ~10% of experimental responses. This rate of response anticipation is likely an underestimation since we were limited by the availability of relevant profile motifs for predicting peptide-HLA I binding (detailed in Material and Methods). Thus, we could only predict peptide-HLA I binding for 8 out of 19 HLA I alleles expressed by the five test subjects detailed in Table 2. In any case, this rate for anticipating response is actually within the reported epitope discovery rate (10), and it is clear that most experimental responses can be predicted. Likewise, we found that positive responses represent a minority of predicted responses (Figure 4). In other words, we predict far more responses than we actually detected. The number of detected responses ranged from 11 out of 55 predicted for A^*^02:01 to none out of 22 for B^*^51:01. It is worth mention that the number of predicted responses is linked to both the number of peptides predicted to bind a given HLA I molecule and the number of subjects who expressed that HLA molecule. Thus, the number of predicted A^*^02:01 responses is larger than that for all the other HLA I molecules because all the test subjects were typed positive for A^*^02:01.

## Discussion

Using T cell reactivity against human cytomegalovirus (HCMV) Epstein-Barr virus (EBV) and influenza as prototypical examples, we present data confirming that the epitope space of these viruses comprises numerous antigens. Moreover, these data highlight the variable hierarchy in T cell recognition of these target antigens amongst the subjects we tested (Table 1 and Supplementary Table 2). The notion that T cells target multiple viral antigens without a clear pattern of immunodominance in the human population for an individual antigen might be a generalizable finding, which is likely to apply to many other viruses as well. Therefore, immune monitoring efforts for HCMV, EBV (and possibly most other viruses), that narrows in on a single viral antigen has a high likelihood of misrepresenting the high- or low-responder status of an individual, and even of providing false negative results. Our data call attention to the need for antigen genome-wide immune monitoring efforts.

Assessment of T cell reactivity against a single, well-defined HCMV pp65 peptide (pp65_495−503_) *vs*. a pool of pp65 peptides clearly demonstrated that the single peptide-induced recall response frequently underestimated the magnitude of the expressed T cell repertoire targeting the entire pp65 antigen, and in some instances failed to detect it entirely (Figures 1, 2). Likewise, assessment of T cell reactivity against additional single peptides from the pp65 antigen *vs*. the pp65 peptide pool further reiterated this finding (Figure 3). Similar observations were also made for EBV (Supplementary Figure 2), and for influenza (Supplementary Figure 3). Therefore, relying on single, previously-defined epitopes seems to be insufficient to reliably quantify, or even detect, T cell immunity against these three viruses, and likely against other viruses as well.

Focusing more closely on HCMV-seropositive, HLA-A^*^02:01-expressing test subjects which exhibited discordance between the number of T cells that responded to the “immunodominant” pp65_495−503_ peptide and the pp65 peptide library, we tried to reconcile this discrepancy by testing whether such donors possessed CD8+ T cells that recognized alternative pp65 epitopes. We sought to test this hypothesis through “brute force” epitope scanning of the entire pp65 antigen, using individual 9-mer peptides that span the entire pp65 aa sequence. Indeed, each of these test subjects responded to additional pp65-derived 9-mer peptides and no single 9-mer peptide was universally recognized by each of these donors (Table 2). Taken together, these data further highlight the need to cover epitopes of an antigen comprehensively through usage of peptide libraries, rather than relying on a single or few previously defined “immune dominant” peptides.

One possible solution for performing comprehensive CD8+ T cell immune monitoring is to tailor peptide libraries to specific HLA class I alleles. Existing algorithms can be used to predict likely peptides that encompass the potential epitope space recognized by CD8+ T cells based on HLA binding criteria (28). However, considering each HLA class I allele expressed by individual test subjects, and a complex antigen such as pp65, this is a considerable scope. There will be a multitude of predicted peptides that satisfy the imposed HLA binding criteria, and these peptides will in large be different for each donor. As a much simpler alternative to such designed peptide libraries, one can take the “agnostic” brute force approach in which a peptide library is constructed that walks the entire amino acid sequence in steps of single amino acids. Using this latter method, one can conceivably cover the entire antigenic space of a protein, in any human, without the need for customization. This brute force approach can actually identify peptide responses where prediction fails, as only a minority of the responses can actually be predicted, and moreover, only a minority of predicted responses can actually be detected.

For a “brute force” approach, 9-11 aa peptides have been successfully used to detect epitope-specific CD8+ T cells (13, 14, 29). In these studies, similar to the results presented in this communication, frequently just one or two adjacent peptides will elicit a CD8+ T cell recall response (Table 2 and Supplementary Figure 5) (13, 14, 29). This natural law confirmed here, that is dictated by the closed peptide-binding grove of MHC class I molecules, calls into question the use of peptide libraries consisting of peptides longer than 11 aa, and that walk the protein sequence in steps greater than a single aa. Thus, only comprehensive coverage of all potential epitopes can reveal the exact dimensions of the expressed CD8+ T cell repertoire.

Therefore, in theory, testing of peptide libraries consisting of 9–11 aa length with single amino acid overlaps would be the ideal strategy for “agnostic” CD8+ T cell immune monitoring. However, *in praxi*, until recently such an approach would have been considered impractical and prohibitive. This is due to (a) the number of peptides needed for such an approach, (b) the number of primary lymphoid cells (e.g., PBMC) required, (c) the labor involved in such testing, (d) the scope of data analysis and (e) interpretation of the flood of data obtained. As we illustrated in this work through systematic testing of five donors' PBMC responding to 553 single peptides, all tested in one experiment, these limitations no longer apply.

The primary notion set forth in this communication is that genome-wide comprehensive testing of 9–10 amino acid long peptides is both necessary and feasible for CD8+ T cell immune monitoring: restricting it to a few known “immune dominant” peptides is likely to underestimate or even entirely miss the antigen-specific CD8+ cell repertoire. Immune monitoring of CD8+ cells critically depends on the precise use of peptides, while immune monitoring of CD4+ cells can be done without involving peptides, using the entire protein. Our data also support the notion that large scale peptide scans have become feasible for systematic epitope discovery. Major steps have already been undertaken in this direction for CD4 + T cells (30, 31). Because HLA Class II molecules' peptide-binding grooves are open on both ends, they can both accommodate longer peptides, and are tolerant to frame shifts of the peptides' HLA anchor residues. For this reason, one can expect CD4+ cell determinant mapping to be largely comprehensive when the classic approach is taken using longer peptides (e.g., 15–25-mers) that cover the antigen sequence in larger steps (e.g., 5–15 amino acid increments). This classic approach for CD4+ cells would likely miss many or even most CD8+ cell epitopes, however (see Table 1, and Supplementary Figure 5). Systematically covering all possible non-amer peptides for mapping CD8+ T cell determinants multiplies the number of peptides to be tested, and thus the scope of testing. Our data suggest that, even for CD8+ T cells, comprehensive large-scale genome wide epitope discovery is approaching feasibility.

Recent advances in high-throughput peptide synthesis technologies have enabled manufacturers to offer extensive, even full proteome-spanning peptide libraries at sufficient quality and low cost, including the customization of such libraries. Unpurified peptides are suitable for screening purposes because the HLA alleles and TCR present in the PBMC cultures can be expected to select the “right” peptide synthesis variants that primed the T cell responses *in vivo*. Confirming this notion, we found (see Table 2) that in all five donors tested the purified pp65_495−503_ peptide recalled essentially identical numbers of CD8+ T cells as the corresponding unpurified peptide from the 9-mer pp65 peptide series. However, as crude peptides are variable in both their purity level and yield, in general, it is advisable to validate individual epitopes identified in such screening experiments using more stringently purified peptides. Thus, the availability and manufacture of extensive peptide libraries no longer precludes brute force T cell epitope mapping.

The greatest obstacle, and rate-limiting factor, for brute force epitope mapping is the number of PBMC that can be obtained from a single test subject. For testing 553 peptides at 3 × 10^5^ PBMC/well, plus 23 control wells, we required 1.73 × 10^8^ PBMC from each test subject. Up to 5 × 10^8^ PBMC can be obtained by classic venipuncture. As an alternative, which we relied upon, one can readily obtain 2 × 10^10^, or more, PBMC from a human subject in a single leukapheresis draw while depleting that individual of only 1% of his/her white blood cells. With 2 × 10^10^ PBMC, using ELISPOT, one has sufficient cells to test up to 6 × 10^5^ individual peptides!

When ELISPOT assays are performed in 96-well plates, between 0.1 and 1 × 10^6^ PBMC are plated per well. In this cell density range, the SFU counts (e.g., the number of antigen-specific T cells detected) is strictly linear to the number of APC plated (32). In the 96-well format, 1 × 10^5^ PBMC per well is the lowest cell input that yields reliable data. Lower numbers of PBMC in the 96-well assay no longer form a confluent cell layer at the bottom of the well, and thus predictable T cell-APC interactions become disrupted. Recently, 384-well ELISPOT plates have become available. The membrane size on the bottom of a 384-well ELISPOT plate is one-third (not the expected one-fourth) that of the 96-well plate. The 384-well format permits miniaturization of ELISPOT assays to precisely one-third compared to a 96-well plate, whereby plating precisely one-third the numbers of PBMC yields precisely one-third of the SFU counts (32). Thus, epitope mapping experiments could theoretically be performed using 3 × 10^4^ PBMC per well (per peptide). Based on these basic parameters, using 5 × 10^7^ cells (corresponding to 50 mL of blood), one can test 500 individual peptides in a 96-well plate format. Using 2 × 10^10^ PBMC, acquired through leukapheresis, 2 × 10^5^ individual peptides can be tested in the 96-well format with 1 × 10^5^ PBMC per well. The number of peptides that can be tested individually in a 384-well format follows by multiplying the above numbers by 3, that is, using the 384-well format, as many as 6 × 10^5^ individual peptides can be tested against PBMC from a donor following a single leukapheresis. Thus, similar to the number of peptides, the number of PBMC required for large scale brute force epitope mapping is not an insurmountable limitation.

Testing of large numbers of peptides in a T cell assay requires well-developed logistics. In our case, the peptides were delivered by the manufacturer as powder in the desired well format spread across six individual 96-well plates. The peptides were then dissolved “en block” using a 96-well multichannel pipettor. In this way, master peptide plates were conveniently created from which the peptides were then transferred, again “en block,” into the actual assay plates. Using such a strategy, both the dilution and plating of peptides was fast and fail-proof since the peptide layouts of the master plates were preserved. The PBMC were also added using the 96-well multichannel pipettor to conclude the most labor-intensive components of the assay: the cell culture setup. After a 24 h *in vitro* incubation, during which peptide-specific T cells became activated and secreted IFN-γ following interaction with APC, the 96-well plates were washed to remove cellular material, and detection reagents were added to begin development of the assay, all done by 96-well pipetting.

Using the approach outlined above, the setup of the experiment summarized in Table 2, in which 553 individual peptides plus controls were tested individually on five test subjects, took three investigators ~3 h of work to accomplish. Using this “en block” pipetting approach, theoretically, even the extreme of testing 6 × 10^5^ peptides in 384-well format, requiring 1,563 plates, could reasonably be achieved with the assistance of pipetting robots.

As illustrated by the data presented herein, epitope recognition by T cells typically involves considerable inter-individual variability, even within HLA-allele matched donor cohorts. Therefore, systematic study of the recognized epitope space of a complex antigen system, such as a virus, will likely require testing of sizable cohorts. Because of the ease of peptide and PBMC plating when the above logistics are followed, PBMC of several donors can be tested in a single experiment. Therefore, performing the T cell ELISPOT assay itself is also not a rate limiting factor for high-throughput, brute force epitope mapping.

The logistics of reading and analyzing ELISPOT data generated in high-throughput epitope mapping experiments involves linking peptide and test subject identities with the experimental data itself. This can be accomplished using the SpotMap™ software, which permits assignment of individual peptides and PBMC donors to specific wells of 96- or 384- well plates, including usage of unique, barcode-based plate identifiers. Using this approach, the specific plate layout can be carried through from peptide synthesis, peptide transfer, plate reading and data analysis. If done in this manner, acquisition and analysis of the raw data, including assignment of positive wells and identification of the specific peptide responsible for cytokine production, is streamlined and in large fully automatic. With the assistance of a plate stacker for the thirty 96-well plates thirty 96-well plates required to generated the data presented in Table 2, the scanning, counting and analysis of the 14,400 wells (corresponding to 14,400 test conditions/data point), took 40 min of fully automatic reader time. With this analysis process progressing at a rate of 1 min per 96-well plate, even the “herculean” task of analyzing the results of testing 600,000 peptides in 1,563 384-well assay plates could be accomplished within 26 h of automated reader time.

## Conclusion

The data presented in this report not only affirm the need for experimental epitope verification, but also serve to make the point that such experiments have become technically feasible. Testing of 553 individual peptides, using five PBMC donors in a single experiment, as reported herein, represents a milestone accomplishment in that direction. We would suggest that, using the technology presented here, it should be possible to epitope map entire genomes, e.g., of viruses. Thereby, for the first time, it will become feasible to study expressed T cell repertoires recognizing entire antigenic systems. In the coming years, we anticipate that such high-resolution studies of individuals', and cohorts', epitope space should become a reality, and will fertilize the field of “epitomics.” Similar to other recently-developed “omics” platforms, the abundance and speed with which new information will be acquired, while overwhelming to the human mind, is destined to shed unprecedented insight into T cell epitope recognition in the context of both health and disease, and eventually will permit the precise assessment of the antigen-specific T cell repertoire as required for accurate immune monitoring of CD8+ T cell immunity.

## Ethics Statement

The white blood cells used in this study were collected under an IRB of Hemacare, Van Nuys, California. They were sold anonymously to CTL, that is CTL, and the authors have no knowledge of the identity of these PBMC donors. Under such conditions, the third party (i.e., CTL and the authors) does not need an IRB to use such cells for research purposes.

## Author Contributions

Experiments were designed by PL, AK, and PR. Experimental data were generated by MS, TZ, DR, GK, and AL, and peptide-binding analysis was performed by PR. PL, GK, and PR prepared the manuscript.

### Conflict of Interest Statement

PV, MS, TZ, DR, GK, AK, and AL are employees of Cellular Technology Limited (CTL), a company that specializes in ELISPOT testing, producing high-throughput-suitable readers, test kits, and GLP-compliant contract research. The remaining author declares that the research was conducted in the absence of any commercial or financial relationships that could be construed as a potential conflict of interest.
