# Supplementary material for: Comprehensive Evaluation of the Expressed CD8+ T Cell Epitope Space Using High-Throughput Epitope Mapping
Source: Front Immunol. 2019 Apr 26;10:655. doi: 10.3389/fimmu.2019.00655 (PMC6499037; doi:10.3389/fimmu.2019.00655)
Supplement: Supplementary file 1 [file Table_1.pdf]

Supplementary Table 1. Demographics and HLA typing of PBMC donors studied

Donor ID	Ethnicity	Age	Gender	HLA-A	HLA-A	HLA-B	HLA-B	HLA-C	HLA-C
1	Hispanic	36	Female	A*68:02	A*68:05/20	B*08:01	B*35:01	C*04:01	C*07:01
2	Caucasian	54	Female	A*02:01	A*33:01	B*14:02	B*27:05	C*01:02	C*08:02
3	Caucasian	50	Female	A*02:01	A*26:01	B*35:03	B*38:01	C*12:03	C*12:01
4	Caucasian	40	Male	A*02:01	A*02:01	B*40:01	B*49:01	C*03:04	C*07:01
6	Caucasian	44	Male	A*02:01	A*24:02	B*07:02	B*13:02	C*06:02	C*07:02
7	Hispanic	33	Male	A*02:01	A*29:02	B*35:01	B*44:03	C*04:01	C*16:01
8	Caucasian	49	Female	A*02:01	A*02:05	B*18:01	B*45:04	C*06:02	C*07:01
9	Caucasian	45	Male	A*02:01	A*03:01	B*44:02	B*44:02	C*05:01	C*05:01
12	Caucasian	36	Male	A*02:01	A*02:01	B*40:01	B*44:02/33	C*03:04	C*05:01
13	Caucasian	44	Male	A*02:01	A*02:01	B*44:02	B*49:01	C*05:01	C*07:01
15	Hispanic	32	Male	A*02:11/12/13	A*30:02	B*39:05	B*44:03	C*07:02	C*16:01
16	Hispanic	24	Male	A*02:01	A*25:01	B*35:01	B*44:03	C*04:01	C*16:01
17	Caucasian	45	Male	A*02:01	A*02:01	B*15:03/64	B*18:01	C*07:01	C*12:03
19	Caucasian	23	Male	A*02:01	A*32:01	B*15:01	B*44:02	C*03:03	C*07:04
20	Caucasian	28	Male	A*02:05	A*24:02	B*08:01	B*50:01	C*06:02	C*07:01
21	Hispanic	18	Male	A*02:06	A*26:01	B*40:02	B*44:03	C*03:04	C*04:01
22	African/American	49	Male	A*01:01	A*68:01	B*57:01	B*57:03/09	C*07:01	C*07:18
23	Caucasian	36	Male	A*02:01	A*29:02	B*08:01	B*40:01	C*03:04	C*07:01
25	Caucasian	52	Male	A*02:01	A*11:01	B*07:02	B*35:01	C*04:01	C*07:02
26	Caucasian	39	Male	A*01:01	A*02:01	B*08:01	B*40:02	C*02:02	C*07:04
27	Caucasian	49	Male	A*02:01	A*24:07	B*40:02	B*44:02	C*05:01	C*15:02
28	Caucasian	46	Female	A*01:01	A*01:01	B*14:02	B*41:02	C*07:01	C*17:01G
31	Caucasian	52	Male	A*02:01	A*11:01	B*07:02	B*35:01	C*04:01	C*07:02
32	Hispanic	61	Male	A*02:01	A*02:05	B*15:01	B*35:01	C*01:02	C*04:01
33	Caucasian	52	Male	A*02:01	A*03:01	B*07:02	B*08:01	C*07:01	C*07:02
34	Hispanic	30	Male	A*02:01	A*02:06	B*08:01	B*40:02	C*07:01	C*15:02
35	Caucasian	24	Male	A*02:01	A*03:01	B*40:01	B*44:02	C*03:04	C*05:01
36	Caucasian	67	Male	A*02:01	A*11:01	B*40:01	B*44:02	C*03:04	C*05:01
37	Hispanic	53	Male	A*02:01	A*34:01	B*38:02	B*40:02	C*07:02	C*15:02
38	Caucasian	32	Male	A*03:01	A*03:01	B*14:02	B*15:01	C*04:01	C*08:02
41	Caucasian	20	Male	A*24:02	A*32:01	B*08:01	B*55:01	C*03:03	C*07:01
42	Caucasian	27	Male	A*02:01	A*03:01	B*07:02	B*27:05	C*01:02	C*07:02
44	Caucasian	38	Male	A*02:01	A*32:01	B*38:01	B*40:02	C*02:02	C*12:03
45	Caucasian	42	Male	A*02:01	A*03:01	B*15:01	B*27:05	C*01:02	C*03:03
46	Caucasian	44	Male	A*02:01	A*68:01	B*07:02	B*44:02	C*07:02	C*07:04
50	Caucasian	55	Female	A*02:01	A*33:01	B*14:02	B*27:05	C*01:02	C*08:02
51	Hispanic	36	Female	A*02:01	A*03:01	B*18:01	B*18:01	C*07:01	C*12:03
52	Hispanic	31	Male	A*02:01	A*02:06	B*08:01	B*40:02	C*07:01	C*15:02
53	Asian	36	Male	A*04:05	A*12:02	B*15:02	B*51:01	C*08:01	C*14:02
56	Caucasian	45	Male	A*02:01	A*68:01	B*07:02	B*44:02	C*07:02	C*07:04
57	Caucasian	54	Male	A*02:01	A*03:01	B*07:02	B*44:02	C*05:01	C*07:02
58	Hispanic	54	Female	A*02:01	A*02:01	B*44:02	B*48:01	C*05:01	C*08:01
60	Caucasian	52	Male	A*02:01	A*33:01	B*35:01	B*44:02	C*04:01	C*05:01
64	Caucasian	48	Female	A*01:01	A*01:01	B*14:02	B*41:02	C*07:01	C*17:03
65	Hispanic	35	Male	A*29:02	A*68:01	B*40:08	B*44:03	C*03:04	C*16:01
70	Caucasian	46	Male	A*02:01	A*02:01	B*13:02	B*18:04	C*06:02	C*12:03
85	African/American	40	Male	A*03:01	A*74:01	B*15:03	B*18:01	C*02:10	C*15:05
89	Caucasian	46	Female	A*01:01	A*02:01	B*39:01	B*40:01	C*03:04	C*07:02
92	Hispanic	28	Male	A*02:01	A*29:02	B*40:01	B*44:03	C*03:04	C*16:01
99	Hispanic	51	Female	A*33:01	A*68:01	B*14:02	B*48:01	C*08:01	C*08:02
100	African/American	36	Male	A*02:01	A*74:01	B*49:01	B*57:03	C*07:01	C*07:01
102	Caucasian	29	Female	A*02:01	A*31:01	B*14:02	B*40:01	C*03:04	C*08:02
111	Hispanic	36	Male	A*02:01	A*68:02	B*40:02	B*81:01	C*03:06	C*18:01
112	Caucasian/Hispanic	44	Female	A*02:01	A*02:05	B*07:02	B*35:03	C*04:01	C*07:02
126	Caucasian	21	Male	A*02:01	A*03:01	B*38:01	B*39:01	C*07:02	C*12:03
130	African/American	54	Male	A*30:02	A*30:02	B*14:02	B*39:10	C*07:18	C*08:02
132	African/American	35	Male	A*02:01	A*03:01	B*57:04	B*58:02	C*06:02P	C*18:02
134	African/American	31	Male	A*02:05	A*30:01	B*42:01	B*58:01	C*07:18	C*17:01

137	African/American	29	Female	A*29:02	A*74:01	B*44:03	B*57:03	C*07:01	C*16:01
141	Hispanic	23	Female	A*24:02	A*33:01	B*14:02	B*14:02	C*02:02	C*08:02
144	Caucasian	43	Male	A*02:01	A*33:03	B*40:01	B*49:01	C*03:04	C*07:01
145	Hispanic	33	Male	A*02:01	A*24:02	B*40:01	B*40:02	C*03:04	C*03:06
151	Hispanic	20	Male	A*02:06	A*68:02	B*15:55	B*48:01	C*04:07	C*08:01
152	Caucasian	45	Female	A*02:01	A*29:02	B*38:01	B*44:02	C*05:01	C*12:03
158	Caucasian	41	Male	A*24:02	A*32:01	B*40:02	B*44:02	C*01:02	C*07:01
159	Filipino	26	Male	A*02:01	A*24:07	B*15:13	B*35:05	C*04:01	C*08:01
167	Asian	38	Male	A*02:01	A*11:01	B*15:13	B*51:01	C*04:01	C*08:01
178	Hispanic	44	Male	A*24:02	A*68:01	B*40:11	B*44:02	C*03:04	C*05:01
182	Caucasian	42	Male	A*29:02	A*68:01	B*39:06	B*40:02	C*03:05	C*07:02
183	Hispanic/Latino	27	Male	A*02:01	A*29:02	B*07:02	B*55:01	C*03:03	C*07:02
192	Hispanic	42	Female	A*02:01	A*24:02	B*15:30	B*40:02	C*01:02	C*03:04
194	Hispanic	27	Female	A*24:02	A*24:02	B*37:01	B*39:06	C*06:02	C*07:02
195	Caucasian	77	Male	A*02:01	A*30:01	B*13:02	B*14:02	C*06:02	C*08:02
199	Hispanic	18	Female	A*02:01	A*31:01	B*35:12	B*40:05	C*04:01	C*07:02
213	Hispanic	52	Male	A*02:01	A*02:06	B*35:01	B*48:01	C*04:01	C*08:01
218	Caucasian	58	Male	A*01:01	A*24:02	B*15:17	B*35:02	C*04:01	C*07:01
221	Caucasian	48	Male	A*02:01	A*02:01	B*40:01	B*49:01	C*03:04	C*07:01
224	Hispanic	35	Male	A*03:01	A*24:02	B*15:10	B*35:01	C*03:04	C*04:01
227	Hispanic	31	Female	A*02:01	A*02:06	B*35:02	B*39:01	C*04:01	C*12:03
228	Hispanic	20	Male	A*02:01	A*02:06	B*35:12	B*40:02	C*03:06	C*04:01
233	African/American	40	Female	A*02:01	A*38:02	B*42:01	B*52:01	C*12:02	C*17:01G
240	Hispanic	24	Male	A*01:01	A*02:01	B*38:01	B*53:01	C*06:02	C*12:03
241	Caucasian	48	Female	A*02:01	A*24:02	B*40:01	B*40:01	C*03:04	C*07:02
251	African/American	20	Male	A*02:01	A*03:01	B*07:02	B*82:02	C*02:10	C*07:02
254	Caucasian	37	Male	A*02:01	A*02:01	B*15:01	B*15:247	C*03:03	C*05:01
261	Asian	41	Male	A*02:01	A*26:01	B*40:06	B*54:01	C*01:02	C*03:04
263	Caucasian	57	Male	A*02:01	A*03:01	B*07:02	B*08:01	C*07:01	C*07:02
264	Hispanic/Latino	24	Male	A*02:01	A*02:06	B*44:02	B*48:03	C*05:01	C*08:01
269	Caucasian	43	Female	A*01:01	A*02:01	B*35:03	B*40:01	C*03:04	C*04:01
278	Hispanic/Latino	26	Male	A*02:01	A*02:05	B*49:01	B*50:01	C*06:02	C*07:01
279	Hispanic/Latino	28	Male	A*02:01	A*68:02	B*51:01	B*53:01	C*04:01	C*15:09
281	Hispanic/Latino	28	Male	A*02:01	A*25:01	B*45:01	B*51:01	C*15:02	C*16:01
284	Hispanic/Latino	36	Male	A*02:01	A*68:01	B*40:08	B*44:03	C*03:04	C*16:01
285	Hispanic/Latino	22	Female	A*02:01	A*03:01	B*35:01	B*45:01	C*06:02	C*16:01
299	Caucasian	40	Male	A*01:01	A*02:01	B*18:04	B*51:01	C*02:02	C*12:03
300	Hispanic	30	Male	A*02:01	A*24:02	B*39:05	B*51:01	C*02:02	C*07:02
303	Hispanic	38	Male	A*02:01	A*31:01	B*14:02	B*51:01	C*01:02	C*08:02
307	Hispanic	22	Female	A*02:01	A*02:06	B*39:02	B*39:05	C*03:04	C*07:02
309	Caucasian/Hispanic	41	Male	A*02:01	A*11:01	B*35:01	B*48:01	C*04:01	C*08:01
312	Hispanic	18	Male	A*02:01	A*02:01	B*07:02	B*35:17	C*04:01	C*07:02
316	Caucasian	25	Male	A*02:01	A*23:01	B*27:05	B*58:01	C*01:02	C*07:01
331	Hispanic	40	Male	A*02:01	A*68:03	B*39:05	B*51:01	C*07:02	C*15:09
350	Caucasian	71	Male	A*02:01	A*03:01	B*07:24	B*18:01	C*07:01	C*07:02
