# Supplementary material for: Comprehensive Evaluation of the Expressed CD8+ T Cell Epitope Space Using High-Throughput Epitope Mapping
Source: Front Immunol. 2019 Apr 26;10:655. doi: 10.3389/fimmu.2019.00655 (PMC6499037; doi:10.3389/fimmu.2019.00655)
Supplement: Supplementary file 2 [file Table_2.pdf]

| Donor ID# | | Medium | EBV antigens / peptide pools (No. of peptides) | | | | | | | | | | | | | |
|---|---|---|---|---|---|---|---|---|---|---|---|---|---|---|---|---|
| | | | BMLF1(117)[1] | BMRF1(99)[2] | BRLF1(149)[3] | BZLF1(59)[4] | EBNA-LP(124)[5] | EBNA1(158)[6] | EBNA2(119)[7] | EBNA3a(234)[8] | EBNA3b(279)[9] | EBNA3c(265)[10] | GP350(224)[11] | LMP1(94)[12] | LMP2(122)[13] | Positive control CPI Pool |
| EBV seropositive donors | 99 | 2 | 3 | 7 | 3 | 3 | 133 | 353 | 20 | 13 | 47 | 403 | 17 | 0 | 7 | 2073 |
| | 194 | 0 | 33 | 7 | 320 | 27 | 3 | 57 | 270 | 33 | 33 | 27 | 17 | 7 | 0 | 655 |
| | 224 | 3 | 83 | 473 | 117 | 1707 | 180 | 1193 | 517 | 163 | 557 | 233 | 60 | 13 | 57 | 2185 |
| | 130 | 0 | 3 | 0 | 27 | 517 | 83 | 203 | 43 | 867 | 33 | 7 | 143 | 0 | 0 | 578 |
| | 85 | 3 | 17 | 3 | 0 | 87 | 0 | 7 | 0 | 433 | 7 | 13 | 10 | 0 | 3 | 553 |
| | 178 | 0 | 23 | 63 | 283 | 13 | 20 | 313 | 187 | 17 | 7 | 233 | 37 | 10 | 577 | 1048 |
| | 134 | 17 | 37 | 220 | 83 | 183 | 60 | 110 | 200 | 20 | 183 | 47 | 3 | 0 | 30 | 280 |
| | 182 | 17 | 40 | 60 | 127 | 243 | 213 | 200 | 1783 | 120 | 53 | 53 | 67 | 13 | 123 | 1738 |
| | 218 | 0 | 20 | 60 | 223 | 30 | 27 | 140 | 243 | 200 | 73 | 110 | 30 | 0 | 360 | 665 |
| EBV seronegative donors | 53 | 7 | 0 | 10 | 10 | 0 | 17 | 13 | 0 | 0 | 7 | 3 | 0 | 3 | 7 | 493 |
| | 7 | 40 | 0 | 13 | 13 | 0 | 3 | 23 | 27 | 0 | 0 | 7 | 0 | 3 | 7 | 443 |
| | 92 | 2 | 13 | 10 | 23 | 3 | 7 | 13 | 0 | 0 | 13 | 23 | 3 | 0 | 0 | 1503 |
| | 158 | 10 | 13 | 17 | 0 | 3 | 10 | 0 | 13 | 0 | 7 | 0 | 7 | 7 | 7 | 1038 |
| | 89 | 3 | 10 | 0 | 0 | 3 | 30 | 3 | 37 | 20 | 3 | 27 | 0 | 0 | 0 | 733 |

**Supplementary Table 2.** EBV-seropositive and seronegative healthy human donors' PBMC responding to the specified EBV antigens. * All results are expressed as SFU/1x$10^6$ PBMC tested per well;legend to Table 1 applies

[1]mRNA export factor EB2 (BMLF1) of EBV; [2]DNA polymerase processivity factor BMRF1 of EBV; [3]Transcription activator BRLF1 of EBV; [4]Trans-activator protein BZLF1 of EBV; [5]antigen leader protein of EBV; [6]nuclear antigen 1 of EBV; [7]nuclear antigen 2 of EBV; [8]nuclear antigen 3 of EBV; [9]nuclear antigen 4 of EBV; [10]nuclear antigen 6 of EBV; [11]Envelope glycoprotein GP350/GP340 of EBV; [12]Latent memebrane protein 1 of EBV; [13]Latent membrane protein 2 of EBV
