# Supplementary material for: Comprehensive Evaluation of the Expressed CD8+ T Cell Epitope Space Using High-Throughput Epitope Mapping
Source: Front Immunol. 2019 Apr 26;10:655. doi: 10.3389/fimmu.2019.00655 (PMC6499037; doi:10.3389/fimmu.2019.00655)
Supplement: Supplementary file 3 [file Table_3.pdf]

Supplementary Table 3. Raw data with donors' IDs for Figure 1

Fig 1A

Donor ID	Medium	pp65(495-503)	CMVpp65 peptide pool
8	1	2	0
23	6	3	7
31	5	0	2
56	0	3	8
70	3	0	7
89	3	0	12
92	1	0	0
102	0	2	3
112	4	3	2
126	3	7	2
145	4	0	3
159	3	5	3
167	0	0	2
199	0	0	0
269	0	2	2
312	4	1	4

Fig 1B

Donor ID	pp65(495-503)	CMVpp65 peptide pool
26	2	47
100	72	138
111	28	327
132	37	387
144	40	532
152	925	1500
183	8	507
192	408	572
195	257	320
213	170	792
221	1500	1500
227	366	588
228	59	249
233	53	100
240	63	227
241	240	533
251	0	150
254	322	370
261	1370	1487
263	160	1500
264	1400	1485
278	689	680
279	479	571
281	35	105
284	99	423
285	15	239
299	33	518
300	350	529
303	366	571
307	74	469
309	190	1500
316	208	188
