# Supplementary material for: Comprehensive Evaluation of the Expressed CD8+ T Cell Epitope Space Using High-Throughput Epitope Mapping
Source: Front Immunol. 2019 Apr 26;10:655. doi: 10.3389/fimmu.2019.00655 (PMC6499037; doi:10.3389/fimmu.2019.00655)
Supplement: Supplementary file 4 [file Table_4.pdf]

Supplementary Table 4. Raw data with donors' IDs for Figure 2

Fig 2A

CEF #	Virus	Antigen source	Epitope sequence	HLA Restriction
CEF-05	EBV	LMP2A (426–434)	CLGGLLTMV	A2
Donor ID	HLA-A	HLA-A	CEF-5-EBV	
2	A*02:01	A*33:01	347.5	
3	A*02:01	A*26:01	7.5	
4	A*02:01	A*02:01	1947.5	
6	A*02:01	A*24:02	2.5	
7	A*02:01	A*29:02	2.5	
8	A*02:01	A*02:05	2.5	
9	A*02:01	A*03:01	2.5	
12	A*02:01	A*02:01	185	
13	A*02:01	A*02:01	55	
16	A*02:01	A*25:01	7.5	
17	A*02:01	A*02:01	297.5	
19	A*02:01	A*32:01	47.5	
25	A*02:01	A*11:01	7.5	
26	A*01:01	A*02:01	37.5	
27	A*02:01	A*24:07	140	
32	A*02:01	A*02:05	0	
33	A*02:01	A*03:01	2.5	
34	A*02:01	A*02:06	5	
35	A*02:01	A*03:01	62.5	
36	A*02:01	A*11:01	0	
37	A*02:01	A*34:01	0	
42	A*02:01	A*03:01	142.5	
44	A*02:01	A*32:01	35	
45	A*02:01	A*03:01	50	
46	A*02:01	A*68:01	5	
50	A*02:01	A*33:01	7.5	
51	A*02:01	A*03:01	2.5	
52	A*02:01	A*02:06	2.5	
57	A*02:01	A*03:01	12.5	
58	A*02:01	A*02:01	92.5	
60	A*02:01	A*33:01	65	

Fig 2B

CEF #	Virus	Antigen source	Epitope sequence	HLA Restriction
CEF-06	EBV	BMLF1 (259–267)	GLCTLVAML	A2
Donor ID	HLA-A	HLA-A	CEF-6-EBV	
2	A*02:01	A*33:01	32.5	
3	A*02:01	A*26:01	30	
4	A*02:01	A*02:01	42.5	
6	A*02:01	A*24:02	10	
7	A*02:01	A*29:02	2.5	
8	A*02:01	A*02:05	2.5	
9	A*02:01	A*03:01	87.5	
12	A*02:01	A*02:01	1047.5	
13	A*02:01	A*02:01	97.5	
16	A*02:01	A*25:01	12.5	
17	A*02:01	A*02:01	42.5	
19	A*02:01	A*32:01	95	
25	A*02:01	A*11:01	2.5	
26	A*01:01	A*02:01	105	
27	A*02:01	A*24:07	102.5	
32	A*02:01	A*02:05	10	
33	A*02:01	A*03:01	37.5	
34	A*02:01	A*02:06	2.5	
35	A*02:01	A*03:01	50	
36	A*02:01	A*11:01	20	
37	A*02:01	A*34:01	2.5	
42	A*02:01	A*03:01	635	
44	A*02:01	A*32:01	10	
45	A*02:01	A*03:01	12.5	
46	A*02:01	A*68:01	10	
50	A*02:01	A*33:01	7.5	
51	A*02:01	A*03:01	15	
52	A*02:01	A*02:06	2.5	
57	A*02:01	A*03:01	112.5	
58	A*02:01	A*02:01	52.5	
60	A*02:01	A*33:01	5	

Fig 2C

CEF #	Virus	Antigen source	Epitope sequence	HLA Restriction
CEF-11	EBV	BRLF1 (148–156)	RVRAYTYSK	A3
Donor ID	HLA-A	HLA-A	CEF-11-EBV	
9	A*02:01	A*03:01	2.5	
33	A*02:01	A*03:01	2.5	
35	A*02:01	A*03:01	25	
38	A*03:01	A*03:01	22.5	
42	A*02:01	A*03:01	5	
45	A*02:01	A*03:01	5	
51	A*02:01	A*03:01	1072.5	
57	A*02:01	A*03:01	20	

Fig 2D

CEF #	Virus	Antigen source	Epitope sequence	HLA Restriction
CEF-12	EBV	EBNA 3a (603–611)	RLRAEAQVK	A3
Donor ID	HLA-A	HLA-A	CEF-12-EBV	
9	A*02:01	A*03:01	5	
33	A*02:01	A*03:01	5	
35	A*02:01	A*03:01	7.5	
38	A*03:01	A*03:01	7.5	
42	A*02:01	A*03:01	65	
45	A*02:01	A*03:01	17.5	
51	A*02:01	A*03:01	15	
57	A*02:01	A*03:01	10	

Fig 2E

CEF #	Virus	Antigen source	Epitope sequence	HLA Restriction
CEF-14	EBV	EBNA 3b (399–408)	AVFDRKSDAK	A11
Donor ID	HLA-A	HLA-A	CEF-14-EBV	
25	A*02:01	A*11:01	7.5	
36	A*02:01	A*11:01	100	

Fig 2F

CEF #	Virus	Antigen source	Epitope sequence	HLA Restriction
CEF-15	EBV	EBNA 3b (416–424)	IVTDFSVIK	A11
Donor ID	HLA-A	HLA-A	CEF-15-EBV	
25	A*02:01	A*11:01	5	
36	A*02:01	A*11:01	395	
