# Supplementary material for: Comprehensive Evaluation of the Expressed CD8+ T Cell Epitope Space Using High-Throughput Epitope Mapping
Source: Front Immunol. 2019 Apr 26;10:655. doi: 10.3389/fimmu.2019.00655 (PMC6499037; doi:10.3389/fimmu.2019.00655)
Supplement: Supplementary file 5 [file Table_5.pdf]

Supplementary Table 5. Raw data with donors' IDs for Figure 3

3A

CEF #	Virus	Antigen source	Epitope sequence	HLA Restriction
CEF-29	HCMV	pp65 (123–131)	IPSINVHHY	B35

Donor ID	HLA-B	HLA-B	CEF-29-CMV
16	B*35:01	B*44:03	2.5
7	B*35:01	B*44:03	2.5
60	B*35:01	B*44:02	5
25	B*07:02	B*35:01	5
1	B*08:01	B*35:01	865
32	B*15:01	B*35:01	12.5

3B

CEF #	Virus	Antigen source	Epitope sequence	HLA Restriction
CEF-31	HCMV	pp65 (511–525)	EFFWDANDIY	B44

Donor ID	HLA-B	HLA-B	CEF-31-CMV
7	B*35:01	B*44:03	0
9	B*44:02	B*44:02	2.5
12	B*40:01	B*44:02/33	65
13	B*44:02	B*49:01	62.5
15	B*39:05	B*44:03	17.5
16	B*35:01	B*44:03	2.5
19	B*15:01	B*44:02	7.5
21	B*40:02	B*44:03	10
27	B*40:02	B*44:02	340
35	B*40:01	B*44:02	5
36	B*40:01	B*44:02	52.5
46	B*07:02	B*44:02	0
57	B*07:02	B*44:02	17.5
58	B*44:02	B*48:01	5
60	B*35:01	B*44:02	0

3C

CEF #	Virus	Antigen source	Epitope sequence	HLA Restriction
CEF-32	HCMV	pp65 (417–426)	TPRVTGGGAM	B7

Donor ID	HLA-B	HLA-B	CEF-32-CMV
6	B*07:02	B*13:02	5
25	B*07:02	B*35:01	2.5
33	B*07:02	B*08:01	1577.5
42	B*07:02	B*27:05	0
46	B*07:02	B*44:02	0
57	B*07:02	B*44:02	1692.5
