# Supplementary material for: Comprehensive Evaluation of the Expressed CD8+ T Cell Epitope Space Using High-Throughput Epitope Mapping
Source: Front Immunol. 2019 Apr 26;10:655. doi: 10.3389/fimmu.2019.00655 (PMC6499037; doi:10.3389/fimmu.2019.00655)
Supplement: Supplementary file 6 [file Table_6.pdf]

Supplementary Table 6. Raw data with donors' IDs for Figure 3

Fig 3A

CEF #	Virus	Antigen source	Epitope sequence	HLA Restriction
CEF-01	Influenza	PB1 (591–599)	VSDGGPNLY	A1
Donor ID	HLA-A	HLA-A	CEF-1-Flu	
22	A*01:01	A*68:01	22.5	
26	A*01:01	A*02:01	5	
28	A*01:01	A*01:01	12.5	

Fig 3B

CEF #	Virus	Antigen source	Epitope sequence	HLA Restriction
CEF-02	Influenza	NP(44–52)	CTELKLSDY	A1
Donor ID	HLA-A	HLA-A	CEF-2-Flu	
22	A*01:01	A*68:01	255	
26	A*01:01	A*02:01	7.5	
28	A*01:01	A*01:01	62.5	

Fig 3C

CEF #	Virus	Antigen source	Epitope sequence	HLA Restriction
CEF-03	Influenza	M1 (58–66)	GILGFVFTL	A2
Donor ID	HLA-A	HLA-A	CEF-3-Flu	
2	A*02:01	A*33:01	10	
3	A*02:01	A*26:01	100	
4	A*02:01	A*02:01	180	
6	A*02:01	A*24:02	82.5	
7	A*02:01	A*29:02	7.5	
8	A*02:01	A*02:05	20	
9	A*02:01	A*03:01	10	
12	A*02:01	A*02:01	465	
13	A*02:01	A*02:01	22.5	
16	A*02:01	A*25:01	112.5	
17	A*02:01	A*02:01	555	
19	A*02:01	A*32:01	122.5	
25	A*02:01	A*11:01	315	
26	A*01:01	A*02:01	200	
27	A*02:01	A*24:07	92.5	
32	A*02:01	A*02:05	55	
33	A*02:01	A*03:01	22.5	
34	A*02:01	A*02:06	15	
35	A*02:01	A*03:01	152.5	
36	A*02:01	A*11:01	565	
37	A*02:01	A*34:01	120	
42	A*02:01	A*03:01	12.5	
44	A*02:01	A*32:01	117.5	
45	A*02:01	A*03:01	5	
46	A*02:01	A*68:01	37.5	
50	A*02:01	A*33:01	22.5	
51	A*02:01	A*03:01	12.5	
52	A*02:01	A*02:06	165	
57	A*02:01	A*03:01	32.5	
58	A*02:01	A*02:01	30	
60	A*02:01	A*33:01	327.5	

Fig 3D

CEF #	Virus	Antigen source	Epitope sequence	HLA Restriction
CEF-04	Influenza	PA (46–54)	FMYSDFHFI	A2
Donor ID	HLA-A	HLA-A	CEF-4-Flu	
2	A*02:01	A*33:01	60	
3	A*02:01	A*26:01	92.5	
4	A*02:01	A*02:01	1485	
6	A*02:01	A*24:02	80	
7	A*02:01	A*29:02	12.5	
8	A*02:01	A*02:05	20	
9	A*02:01	A*03:01	15	
12	A*02:01	A*02:01	22.5	
13	A*02:01	A*02:01	2.5	
16	A*02:01	A*25:01	5	
17	A*02:01	A*02:01	2.5	
19	A*02:01	A*32:01	7.5	
25	A*02:01	A*11:01	5	
26	A*01:01	A*02:01	5	
27	A*02:01	A*24:07	5	
32	A*02:01	A*02:05	12.5	
33	A*02:01	A*03:01	0	
34	A*02:01	A*02:06	2.5	
35	A*02:01	A*03:01	5	
36	A*02:01	A*11:01	2.5	
37	A*02:01	A*34:01	5	
42	A*02:01	A*03:01	0	
44	A*02:01	A*32:01	2.5	
45	A*02:01	A*03:01	0	
46	A*02:01	A*68:01	7.5	
50	A*02:01	A*33:01	2.5	
51	A*02:01	A*03:01	2.5	
52	A*02:01	A*02:06	5	
57	A*02:01	A*03:01	15	
58	A*02:01	A*02:01	37.5	
60	A*02:01	A*33:01	7.5	

Fig 3E

CEF #	Virus	Antigen source	Epitope sequence	HLA Restriction
CEF-08	Influenza	NP (91–99)	KTGGPIYKR	Aw68
Donor ID	HLA-A	HLA-A	CEF-8-Flu	
22	A*01:01	A*68:01	40	
46	A*02:01	A*68:01	5	

Fig 3F

CEF #	Virus	Antigen source	Epitope sequence	HLA Restriction
CEF-09	Influenza	NP (342–351)	RVLSFIKGTK	A3
Donor ID	HLA-A	HLA-A	CEF-9-Flu	
9	A*02:01	A*03:01	5	
33	A*02:01	A*03:01	0	
35	A*02:01	A*03:01	2.5	
38	A*03:01	A*03:01	7.5	
42	A*02:01	A*03:01	0	
45	A*02:01	A*03:01	7.5	
51	A*02:01	A*03:01	0	
57	A*02:01	A*03:01	2.5	

Fig 3G

CEF #	Virus	Antigen source	Epitope sequence	HLA Restriction
CEF-10	Influenza	NP(265–273)	ILRGSVAHK	A3
Donor ID	HLA-A	HLA-A	CEF-10-Flu	
9	A*02:01	A*03:01	5	
33	A*02:01	A*03:01	0	
35	A*02:01	A*03:01	85	
38	A*03:01	A*03:01	35	
42	A*02:01	A*03:01	20	
45	A*02:01	A*03:01	2.5	
51	A*02:01	A*03:01	60	
57	A*02:01	A*03:01	10	

Fig 3H

CEF #	Virus	Antigen source	Epitope sequence	HLA Restriction
CEF-13	Influenza	M1 (13–21)	SIIPSGPLK	A11
Donor ID	HLA-A	HLA-A	CEF-13-Flu	
25	A*02:01	A*11:01	8	
36	A*02:01	A*11:01	0	

Fig 3I

CEF #	Virus	Antigen source	Epitope sequence	HLA Restriction
CEF-18	Influenza	NP (418–426)	LPFDKTTVM	B7
Donor ID	HLA-B	HLA-B	CEF-18-Flu	
6	B*07:02	B*13:02	5	
25	B*07:02	B*35:01	5	
33	B*07:02	B*08:01	0	
42	B*07:02	B*27:05	0	
46	B*07:02	B*44:02	5	
57	B*07:02	B*44:02	2.5	

Fig 3J

CEF #	Virus	Antigen source	Epitope sequence	HLA Restriction
CEF-20	Influenza	NP (380–388)	ELRSRYWAI	B8
Donor ID	HLA-B	HLA-B	CEF-20-Flu	
1	B*08:01	B*35:01	60	
20	B*08:01	B*50:01	40	
26	B*08:01	B*40:02	77.5	
33	B*07:02	B*08:01	0	
34	B*08:01	B*40:02	57.5	
41	B*08:01	B*55:01	117.5	
52	B*08:01	B*40:02	57.5	

Fig 3K

CEF #	Virus	Antigen source	Epitope sequence	HLA Restriction
CEF-25	Influenza	NP (383–391)	SRYWAIRTR	B27
Donor ID	HLA-B	HLA-B	CEF-25-Flu	
L2	B*14:02	B*27:05	50	
42	B*07:02	B*27:05	95	
45	B*15:01	B*27:05	2.5	
50	B*14:02	B*27:05	10	

Fig 3L

CEF #	Virus	Antigen source	Epitope sequence	HLA Restriction
CEF-26	Influenza	M1 (128–135)	ASCMGLIY	B27
Donor ID	HLA-B	HLA-B	CEF-26-Flu	
L2	B*14:02	B*27:05	25	
42	B*07:02	B*27:05	0	
45	B*15:01	B*27:05	7.5	
50	B*14:02	B*27:05	2.5	
