# Supplementary material for: Comprehensive Evaluation of the Expressed CD8+ T Cell Epitope Space Using High-Throughput Epitope Mapping
Source: Front Immunol. 2019 Apr 26;10:655. doi: 10.3389/fimmu.2019.00655 (PMC6499037; doi:10.3389/fimmu.2019.00655)
Supplement: Supplementary file 7 [file Image_1.pdf]

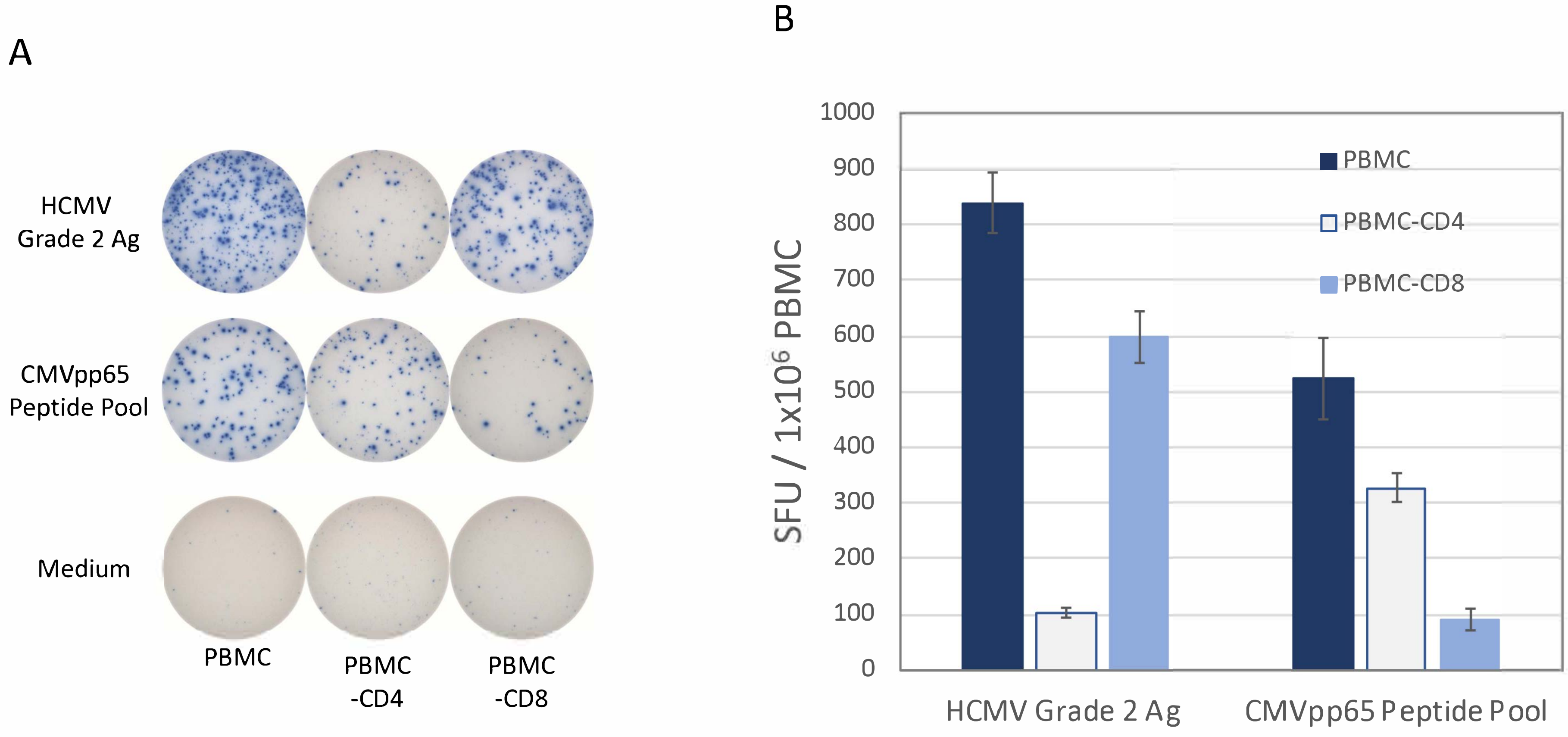

Supplementary Figure 1. The pp65 peptide pool, consisting of 15-mer peptides, recalls both CD4+ and CDB+ T cells. Unfractionated PBMC were tested in IFN-γ EL/SPOT assays, along with the same PBMC following depletion of CD4+ cells {PBMC-CD4} or COB+ cells {PBMC-CDB). These cells were stimulated with UV-inactivated HCMV virions {HCMV Grade 2 Ag) or the 15-mer peptide pool that covers the pp65 aa sequence {CMV pp65 peptide pool}, as specified. Cells cultures in medium alone served as the negative control. (A) Representative wells, and (BJ mean± SD of 3 replicate wells for each condition are shown.
