# Supplementary material for: Comprehensive Evaluation of the Expressed CD8+ T Cell Epitope Space Using High-Throughput Epitope Mapping
Source: Front Immunol. 2019 Apr 26;10:655. doi: 10.3389/fimmu.2019.00655 (PMC6499037; doi:10.3389/fimmu.2019.00655)
Supplement: Supplementary file 8 [file Image_2.pdf]

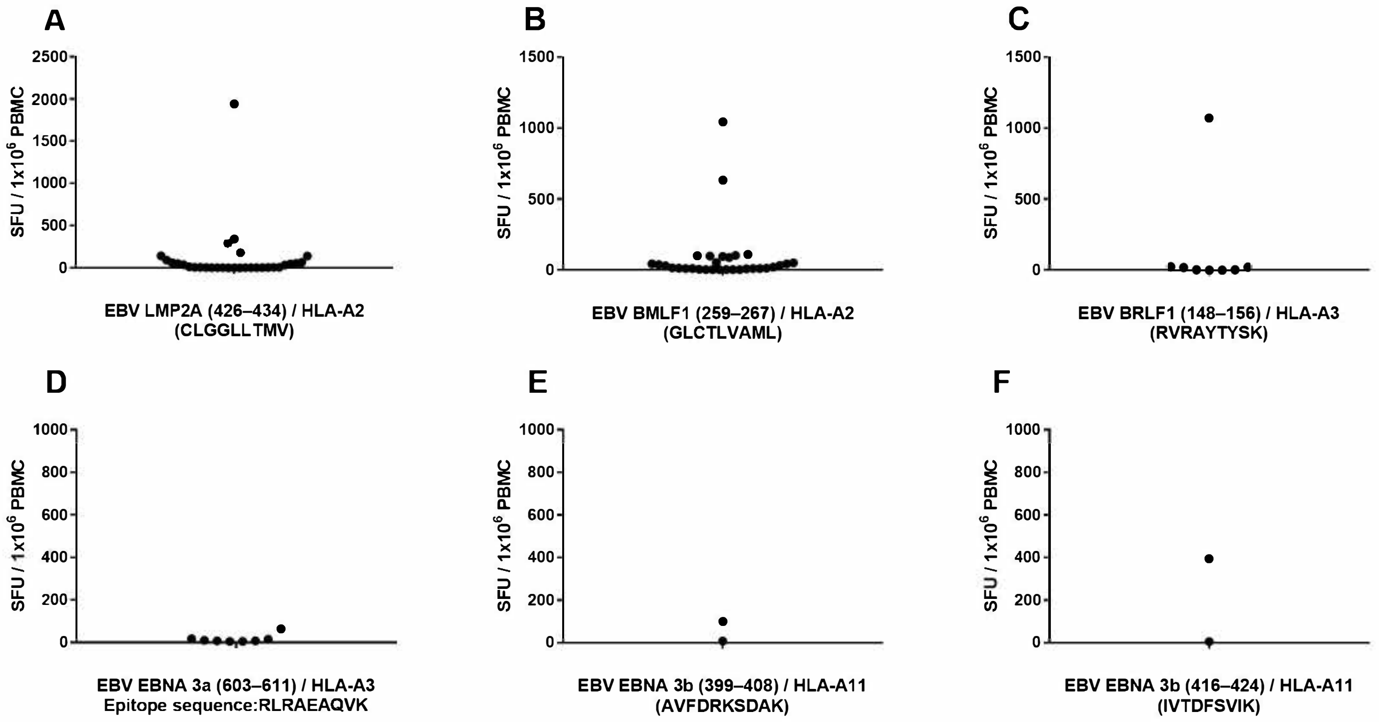

Supplementary Figure 2. CD8+ T cell recognition of previously defined EBV epitopes in PBMC of HLA matched, EBV-seropositive subjects. Test results using the specified peptides (A) LMP $2A_{426\text{-}434}$, (B) $BMLF1_{259\text{-}267}$, (C) $BRLF1_{148\text{-}156}$, (D) EBNA $3a_{603\text{-}611}$, (E) EBNA $3b_{399\text{-}408}$ and (F) EBNA $3B_{416\text{-}424}$ along with the previously defined HLA restriction are indicated. Each data point (dot) within the panels represents the number of IFN-γ spot forming units (SFU) elicited by that peptide in *a* donor bearing the corresponding HLA class I allele. The individual peptides are part of the CEF peptide pool, and the original references are listed in Currier et al., J. Immunol. Methods., 2001, 260:157-172.
