# Supplementary material for: Comprehensive Evaluation of the Expressed CD8+ T Cell Epitope Space Using High-Throughput Epitope Mapping
Source: Front Immunol. 2019 Apr 26;10:655. doi: 10.3389/fimmu.2019.00655 (PMC6499037; doi:10.3389/fimmu.2019.00655)
Supplement: Supplementary file 9 [file Image_3.pdf]

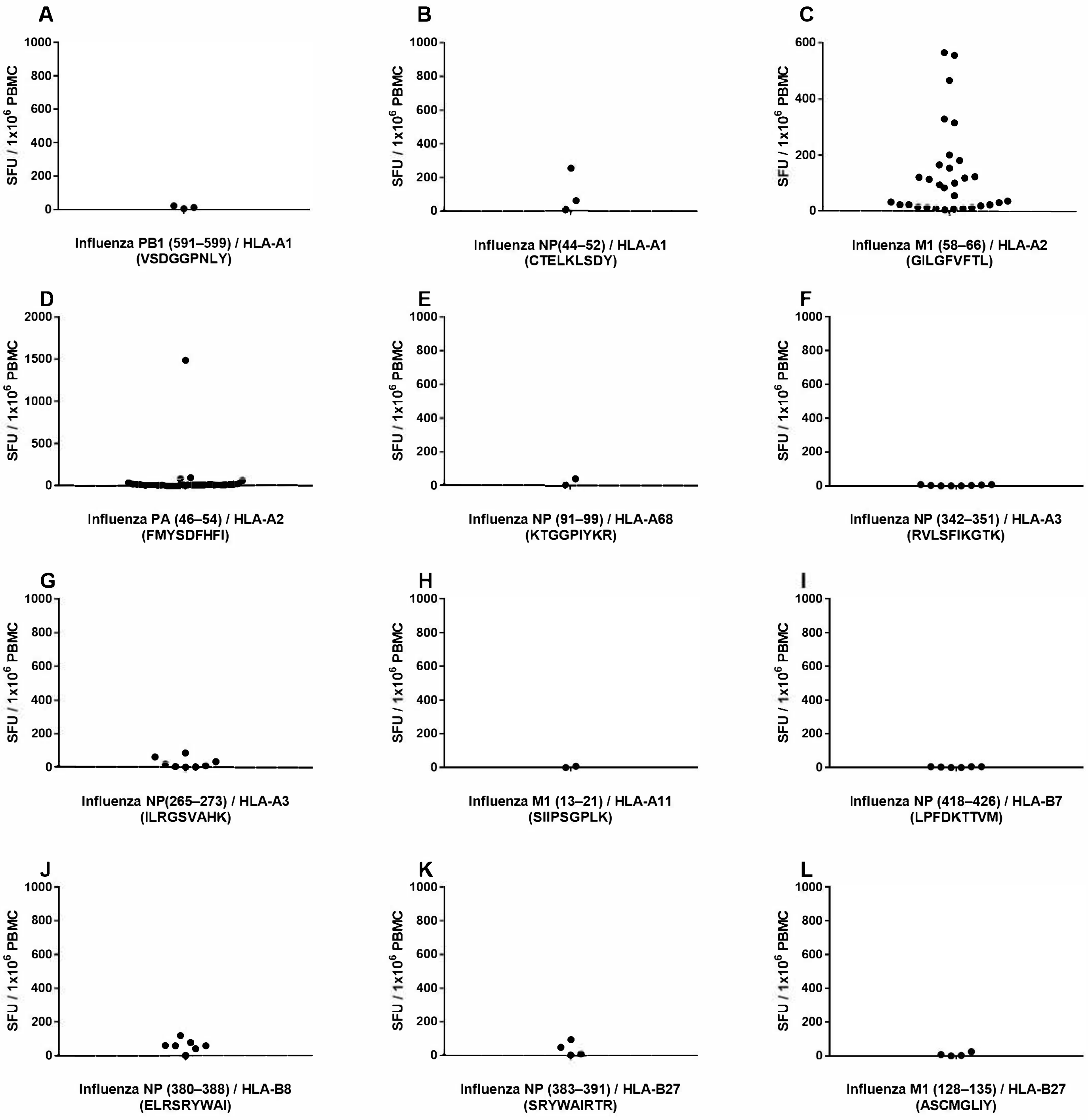


**Supplementary Figure 3.** CD8+ T cell recognition of previously defined influenza virus epitopes in PBMC of HLA matched subjects. Test results using the specified peptides (A) $PB1_{591-599}$ , (B) $NP_{44-52}$, (C) $M1_{58-66}$, (D) $PA_{46-54}$, (E) $NP_{91-99}$, (F) $NP_{342-351}$, (G) $NP_{265-273}$, (H) $M1_{13-21}$, (I) $NP_{418-426}$, (J) $NP_{380-388}$, (K) $NP_{383-391}$ and (L) $M1_{128-135}$ along with the previously defined HLA restriction are indicated. Each data point (dot) within the panel represents the number of IFN-γ SFU elicited by that peptide in a donor bearing the corresponding HLA class I allele. The peptides are part of the CEF peptide pool, and the original references are listed in Currier et al., J. Immunol. Methods., 2001, 260:157-172.
