# Supplementary material for: Comprehensive Evaluation of the Expressed CD8+ T Cell Epitope Space Using High-Throughput Epitope Mapping
Source: Front Immunol. 2019 Apr 26;10:655. doi: 10.3389/fimmu.2019.00655 (PMC6499037; doi:10.3389/fimmu.2019.00655)
Supplement: Supplementary file 10 [file Image_4.pdf]

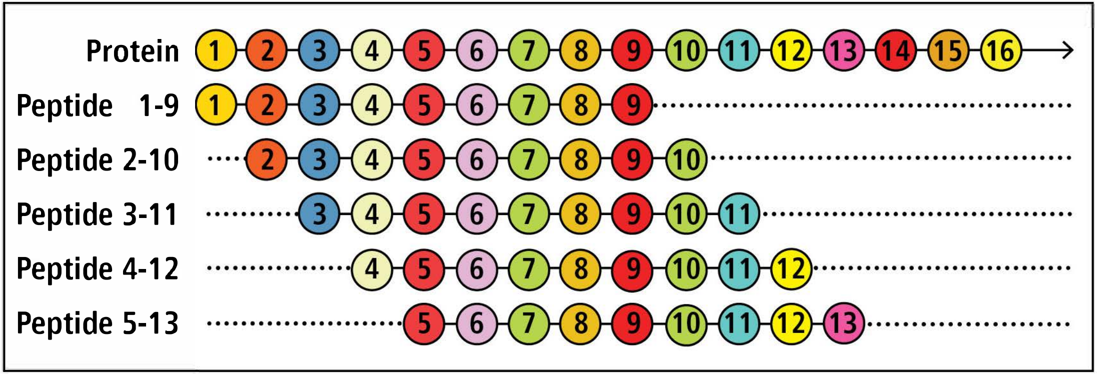

Supplementary Figure 4. Illustration of the 9-mer peptide library used for brute force epitope mapping of HCMV pp65. The first sixteen positions of the pp65 amino acid sequence are shown on the top. The individual peptides walk the sequence in steps of single amino acids.
