# Supplementary material for: Comprehensive Evaluation of the Expressed CD8+ T Cell Epitope Space Using High-Throughput Epitope Mapping
Source: Front Immunol. 2019 Apr 26;10:655. doi: 10.3389/fimmu.2019.00655 (PMC6499037; doi:10.3389/fimmu.2019.00655)
Supplement: Supplementary file 11 [file Image_5.pdf]

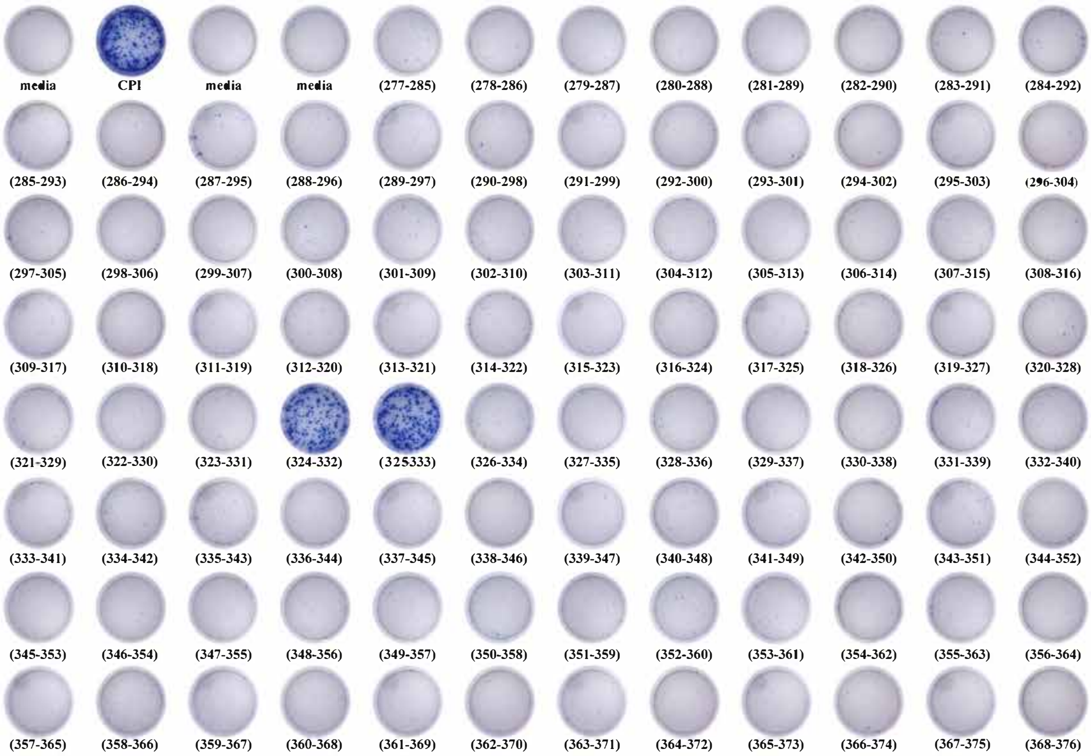

Supplementary Figure 5. Representative example of EL/SPOT-based EpiScan results. Images of each well on a 96 well plate are shown. The antigen used for stimulation is specified underneath each well. Wells A1, A3, and A4 are negative (media) and A2 positive {CPI} control wells respectively. Starting well A5, each well contains the specified 9-mer peptide of the pp65 antigen. Note the SFU formation in wells E4 and E5, elicited by peptides $pp65_{324-332}$ and $pp65_{32S-333}$, respectivelY, identifying a COB cell epitope. Note also the lack of SFU formation in all other wells, except the CPI positive control.
